# Local Release of TGF‐*β* Inhibitor Modulates Tumor‐Associated Neutrophils and Enhances Pancreatic Cancer Response to Combined Irreversible Electroporation and Immunotherapy

**DOI:** 10.1002/advs.202105240

**Published:** 2022-02-07

**Authors:** Huiming Peng, Jian Shen, Xin Long, Xiaoqi Zhou, Jiaqi Zhang, Xina Xu, Teng Huang, Hui Xu, Shuguo Sun, Chun Li, Ping Lei, Heshui Wu, Jun Zhao

**Affiliations:** ^1^ Department of Anatomy School of Basic Medicine Huazhong University of Science and Technology Wuhan Hubei Province 430030 China; ^2^ Department of Pancreatic Surgery Union Hospital Tongji Medical College Huazhong University of Science and Technology Wuhan Hubei Province 430022 China; ^3^ Department of Histology and Embryology School of Basic Medicine Huazhong University of Science and Technology Wuhan Hubei Province 430030 China; ^4^ Department of Immunology School of Basic Medicine Huazhong University of Science and Technology Wuhan Hubei Province 430030 China; ^5^ Ultrastructural Pathology Laboratory Department of Pathology School of Basic Medicine Tongji Medical College Huazhong University of Science and Technology Wuhan Hubei Province 430030 China; ^6^ Department of Cancer Systems Imaging University of Texas MD Anderson Cancer Houston TX 77030 USA; ^7^ Department of Nuclear Medicine and PET Tongji Hospital Tongji Medical College Huazhong University of Science and Technology Wuhan Hubei Province 430030 China; ^8^ Cell Architecture Research Center Huazhong University of Science and Technology Wuhan Hubei Province 430030 China

**Keywords:** immune checkpoint blockade, mesoporous silica nanoparticles, neutrophil polarization, SB525334, TGF‐*β* inhibitor

## Abstract

Pancreatic cancer is a deadly disease with little response to standard therapies. Irreversible electroporation (IRE) has emerged as a novel ablative technique for the clinical treatment of pancreatic cancer. Combinations of IRE and immunotherapies, including anti‐programmed death 1 (*α*PD1) immune checkpoint blockade, have shown promising efficacy in both preclinical and clinical studies. However, tumor recurrence remains an obstacle that needs to be overcome. It herein is shown that IRE induces a substantial infiltration of neutrophils into pancreatic tumors. These neutrophils are then polarized into a protumor phenotype by immunosuppressive cues, in particular transforming growth factor *β* (TGF‐*β*). Using glutathione‐responsive degradable mesoporous silica nanoparticles loaded with SB525334, an inhibitor of TGF‐*β*1 receptor, it is demonstrated that local inhibition of TGF‐*β* within the tumor microenvironment promotes neutrophil polarization into an antitumor phenotype, enhances pancreatic cancer response to combined IRE and *α*PD1 therapy, and induces long‐term antitumor memory. The therapeutic efficacy is also attributed to tumor infiltration by CD8^+^ cytotoxic T cells, depletion of regulatory T cells, and maturation of antigen‐presenting dendritic cells. Thus, modulating neutrophil polarization with nanomedicine is a promising strategy for treating pancreatic cancer.

## Graphical abstract

Treatment of pancreatic tumor by irreversible electroporation (IRE) induces a rapid infiltration by neutrophils, which polarize into a protumor phenotype and negatively correlate with therapeutic outcome. Local release of transforming growth factor‐*β* inhibitor within the tumor microenvironment promotes neutrophils polarization toward an antitumor phenotype and enhances tumor response to combined IRE and immunotherapy.

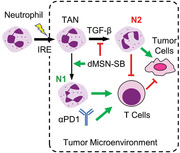

## 1 Introduction

Pancreatic ductal adenocarcinoma (PDAC) is an abysmal disease with a five‐year survival rate below 9%.^[^ ^1^ ^]^ While a majority of patients with PDAC are unfit for surgical resection, they also respond poorly to current chemo‐, radio‐, or immunotherapies because of the fibrotic and immunosuppressive tumor microenvironment of this carcinoma.^[^ ^2^ ^]^ Irreversible electroporation (IRE) is a novel ablative technique used for clinical treatment of patients with PDAC.^[^ ^3^ ^]^ It delivers high‐voltage electric pulses intratumorally to permanently disrupt cell membrane and induce cell death.^[^ ^4^ ^]^ Moreover, IRE has been documented to temporarily alleviate immunosuppression and promote anti‐tumor immunity.^[^ ^5^ ^]^ We previously showed that a combination of IRE and anti‐programmed death 1 (*α*PD1) immune checkpoint blockade significantly improves animal survival in an orthotopic murine PDAC model,^[^ ^6^ ^]^ which corroborated with clinical reports that this combination therapy provides clinical benefits to patients with advanced PDAC.^[^ ^7^ ^]^ Other immunotherapy modalities, such as a toll‐like receptor 7 agonist,^[^ ^8^ ^]^ a nanoformulation of indoleamine‐2,3‐deoxygenase inhibitor,^[^ ^9^ ^]^ and allogenic V*γ*9V*δ*2 T cells,^[^ ^10^ ^]^ also have been shown to synergize with IRE. However, tumor recurrence was still common after the IRE‐based immunotherapies, suggesting that other factors may have also played a role in the therapeutic outcome. In addition, with most of the ongoing research being focused on further stimulation of T‐cell activities, the risk of autoimmune toxicities increases.^[^ ^11^ ^]^

We observed a substantial infiltration of neutrophils into pancreatic tumors after IRE. Neutrophils are the most abundant subpopulation of circulating leukocytes and are the first line of defense against infections.^[^ ^12^ ^]^ The complex roles of tumor‐associated neutrophils (TANs) during tumor progression, however, has only been realized recently.^[^ ^13^ ^]^ TANs can be categorized into the anti‐tumor N1 phenotype and pro‐tumor N2 phenotype.^[^ ^14^ ^]^ Immunosuppressive molecules in the tumor microenvironment, transforming growth factor *β* (TGF‐*β*) in particular, can polarize TANs into the N2 phenotype, which may blunt the anti‐tumor activities of T cells.^[^ ^15^ ^]^ Treatment with TGF‐*β* inhibitors, on the other hand, can shift TANs toward the N1 phenotype and restrain tumor growth.^[^ ^16^ ^]^ As for the IRE‐induced infiltrating neutrophils, it is unknown in which direction they would polarize. It is also unclear whether and how TGF‐*β* inhibition could modulate their polarization.

TGF‐*β* inhibitors like pirfenidone and galunisertib have shown efficacy in clinical and preclinical applications, but their nonspecific accumulation in nontumor organs has been reported to cause side effects.^[^ ^17^ ^]^ Nanomedicine has created a new paradigm for cancer therapy during the previous decades, because of its unique properties such as the integration of multiple theranostic modalities and tumor‐specific drug delivery.^[^ ^18^ ^]^ Specifically, mesoporous silica nanoparticles (MSNs) exhibit several attractive properties including satisfactory biocompatibility and superior capacity for drug loading.^[^ ^19^ ^]^ The incorporation of cleavable linkages also imbued MSNs with accelerated degradability and stimuli‐responsive drug release.^[^ ^20^ ^]^ However, the fibrotic stroma and dysfunctional vasculature of PDAC remain a significant barrier to the intratumoral transport of any nano‐formulations. We herein propose an intratumoral injection of a degradable MSN formulation loaded with TGF‐*β* inhibitor, to minimize drug exposure to other major organs. As this procedure can be performed at the same time of electrode placement for IRE, it is clinically feasible and unlikely to impose any additional burden on patients.

It was our hypothesis that a local release of TGF‐*β* inhibitor within the tumor microenvironment could promote neutrophil polarization into the antitumor N1 phenotype, and enhance pancreatic cancer response to combined IRE and *α*PD1 treatment. To test this hypothesis, we prepared glutathione‐responsive degradable MSNs for highly efficient encapsulation of SB525334, an inhibitor of TGF‐*β*1 receptor, and evaluated the resultant nanoformulation (dMSN‐SB) in the Panc02 murine pancreatic tumor model. The dMSN‐SB nanoparticles successfully disrupted TGF‐*β* signaling and prevented neutrophil polarization toward the protumor N2 phenotype in cell culture. In animal models, we then established that the infiltrating neutrophils shifted toward the N2 phenotype as the tumor recovered from IRE. The triple therapy of IRE, dMSN‐SB, and *α*PD1 promoted TAN polarization toward the antitumor N1 phenotype, significantly increasing survival of tumor‐bearing mice and inducing long‐term anti‐tumor memory. Our findings suggest that TAN modulation is a promising and safe strategy to further enhance pancreatic cancer response to IRE‐based immunotherapy.

## 2 Results

### 2.1 Preparation and Characterization of Degradable Mesoporous Silica Nanoparticles Loaded with SB525334

**Figure 1:**
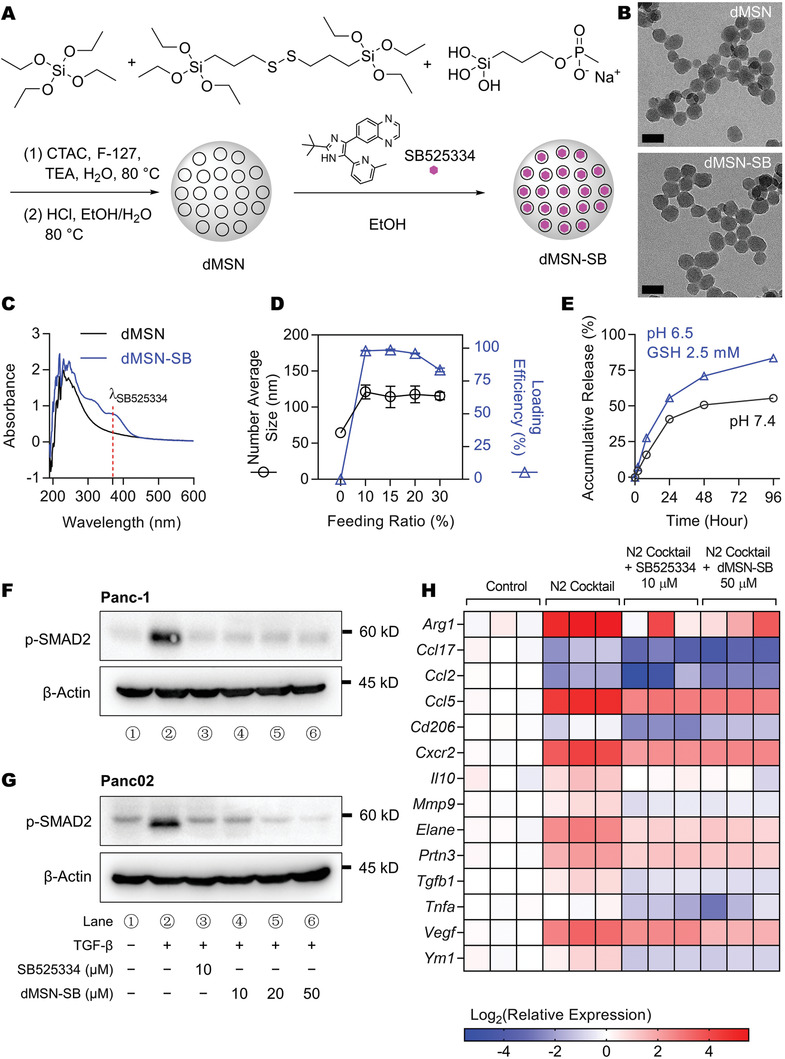
Preparation and characterization of dMSN‐SB. A) Reaction schemes. B) Representative TEM images of dMSN and dMSN‐SB. Scale bar = 50 nm. C) UV–Vis spectra. Absorbance peak of SB525334 was shown at 370 nm. D) Hydrodynamic size and drug loading efficiency of dMSN‐SB at different SB525334‐to‐dMSN feeding ratios. Data are shown as mean ± standard error of mean (SEM), *n* = 3. E) Accumulative release curves of dMSN‐SB at 37 °C in phosphate‐buffered saline (PBS) at pH 7.4 or sodium acetate buffer at pH 6.5 supplemented with 2.5 × 10^‐3^ M GSH. Data are shown as mean ± SEM, *n* = 3. Small error bars covered by the symbol are not shown. F,G) Expression of p‐SMAD2 in F) Panc‐1 human pancreatic cancer cells and G) Panc02 murine pancreatic cancer cells after treatment with TGF‐*β* (20 ng mL^–1^), SB 525334 and/or dMSN‐SB. H) Relative expression of N2‐associated genes in murine bone marrow‐isolated neutrophils after treatment with N2 cocktail, SB525534 (10 × 10^‐6^ m) and/or dMSN‐SB (50 × 10^‐6^ m). Data are shown as heat map with a color scale. Each square represents an individual independent data point. Three replicates were included in each group.

Based on our observation that IRE induced glutathione (GSH) release from cancer cells (Figure S1, Supporting Information), we prepared GSH‐responsive degradable MSNs via sol‐gel method using tetraethyl orthosilicate and bis(triethoxysilylpropyl) disulfide, and coating in situ with 3‐(trihydroxysilyl)propyl methyl phosphonate (**Figure** **1**A) per a previous report with some modifications.^[^ ^21^ ^]^ SB525334 was encapsulated by coincubating with the MSNs in ethanol, and the resultant product (dMSN‐SB) was collected by centrifugation. Transmission electron microscopy (TEM) revealed that both dMSN and dMSN‐SB were spherically shaped with a diameter of 40–50 nm (Figure 1B). Ultraviolet–visible spectroscopy (UV–vis) showed an absorbance peak at 370 nm following loading of SB525334 (Figure 1C). The dMSN nanoparticles exhibited good capacity for loading SB525334, with above 95% loading efficiency for feeding ratios up to 20% by weight. The number‐average hydrodynamic size of dMSN was 64.4 ± 2.5 nm, which increased to about 110 nm after drug loading probably due to particle aggregation (Figure 1D). The dMSN‐SB formulation with a 20% feeding ratio of SB525334 by weight was then used for subsequent studies. SB525334 was released from dMSN in response to acidic pH and GSH, which was relevant to the acidic stromal pH and IRE‐induced GSH release (Figure 1E).

We first examined in vitro whether dMSN‐SB inhibited TGF‐*β* signaling. Both SB525334 (10 × 10^‐6^ m) and dMSN‐SB (10 × 10^‐6^, 20 × 10^‐6^, and 50 × 10^‐6^ m in SB525334 equivalence) inhibited phosphorylation of SMAD2, the downstream target of TGF‐*β* in Panc‐1 (Figure 1F) and Panc02 cells (Figure 1G). A similar inhibition of SMAD2 phosphorylation by dMSN‐SB was also observed in Hs766T human pancreatic cancer cells and 4T1 murine breast cancer cells (Figure S2, Supporting Information). CCK‐8 assay confirmed that dMSN‐SB did not affect cell viability at concentrations up to 200 × 10^‐6^ m in SB525334 equivalent (Figure S3, Supporting Information), suggesting that the TGF‐*β* inhibition was not caused by nonspecific toxicity.

Next, it was examined whether dMSN‐SB modulated neutrophil polarization in vitro. Toward this, naïve neutrophils were isolated from the bone marrow of C57BL/6 mice, and treated with a cocktail of N2‐inducing reagents^[^ ^22^ ^]^ in the absence or presence of dMSN‐SB. Due to the lack of specific surface markers for clear identification of TAN phenotypes, we monitored the mRNA transcription of a panel of N2‐associated genes to comprehensively understand TAN behavior.^[^ ^16^ ^]^ As shown in Figure 1H, the N2 cocktail upregulated mRNA expression in 10 out of 14 N2‐associated genes, suggesting a dramatic shift toward the N2 phenotype. For example, the mRNA expression of *Arg1*, *Ccl5*, *Vegf*, *Cxcr2*, and *Elane* increased by 40, 26, 15, 8, and 6 times, respectively. Adding 50 × 10^‐6^ m dMSN‐SB to the cocktail, on the other hand, mitigated mRNA upregulation in seven N2‐associated genes, and downregulated mRNA expression of the remaining eight genes to levels lower than those of the naïve neutrophils. In the meanwhile, 10 × 10^‐6^ m dMSN‐SB was less effective than 10 × 10^‐6^ m SB525334 in reversing the N2 polarization (Figure S4, Supporting Information), probably due to the incomplete drug release from dMSN‐SB during the 24 h incubation. In summary, our results demonstrated that dMSN‐SB could effectively inhibit TGF‐*β* signaling and prevent neutrophils from polarization into the pro‐tumor N2 phenotype in the cell culture system.

### 2.2 Neutrophils Infiltrated Tumors after IRE and Polarized toward N2 Phenotype

**Figure 2:**
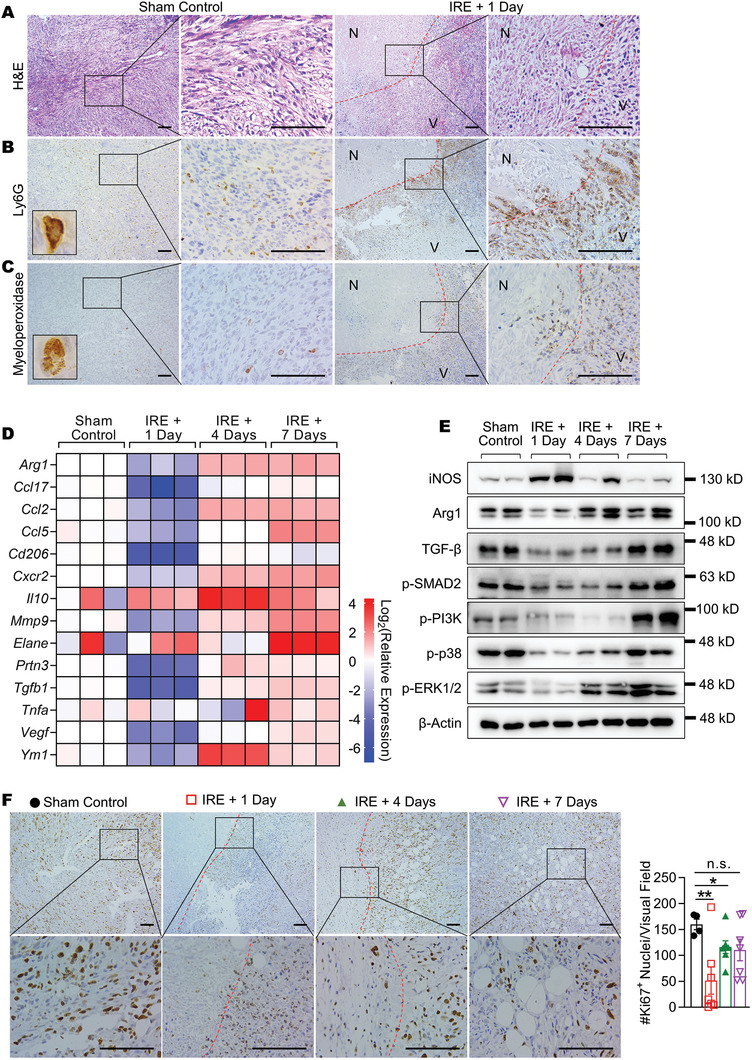
Post‐IRE changes in intratumoral neutrophils and N2‐associated genes. A) H&E, B) Ly6G, and C) myeloperoxidase staining of Panc02 tumors. Borders between the necrotic (N) and viable (V) regions are marked with red dashed lines. Representative images of single Ly6G^+^ or myeloperoxidase^+^ cells are shown in insets. D) Relative expression of N2‐associated genes in Panc02 tumors at different time points post‐IRE. Data are shown as heat map with a color scale. Each square represents an individual independent data point. Three replicates were included in each group. E) Immunoblotting of selective targets in Panc02 tumor lysates. Two tumors were analyzed in each group. F) IHC staining of Ki67 in Panc02 tumor sections. Borders between the necrotic and viable regions were marked with red dashed lines. Scale bar = 100 µm. Four to eight 400× visual fields were imaged at random to quantify Ki67^+^ nuclei. Data are presented as mean ± SEM in bar plots overlaid with individual data points. Significance was determined using one‐way ANOVA followed by Tukey post hoc analysis. *p < 0.05, **p < 0.01, n.s. = not significant.

A substantial infiltration of neutrophils into murine Panc02 tumors was observed on day 1 post‐IRE. Hematoxylin and eosin (H&E) staining revealed a distinct region of IRE‐induced necrosis (**Figure** **2**A). The presence of neutrophils at the border between necrotic and viable regions was verified by the positive staining of two neutrophil biomarkers, Ly6G (Figure 2B) and myeloperoxidase (Figure 2C). The characteristic staining patterns of Ly6G (membrane) and myeloperoxidase (diffuse granules) are shown in figure insets. In contrast, neutrophils were less frequent and more evenly distributed in sham control tumors.

Given that the upregulation of TGF‐*β* signaling is commonly encountered in late‐stage PDAC,^[^ ^23^ ^]^ we postulated that the IRE‐induced infiltrating neutrophils would shift toward the N2 phenotype over time. This postulation was tested in a subcutaneous Panc02 model with a positive TGF‐*β* expression (Figure S5, Supporting Information). Tumors were collected at different time points post‐IRE and analyzed for mRNA expression of fourteen N2‐associated genes. There was a precipitous decrease in mRNA levels among 86% of the genes (12/14) on day 1 post‐IRE (Figure 2D). The mRNA expression of these genes rebounded quickly as the tumor recovered. On day 7 post‐IRE, the mRNA levels in 93% of the genes (13/14) were higher than those in sham control. For example, the mRNA levels of *Arg1* (encoding arginase I), *Elane* (encoding neutrophil elastase), and *Tgfb1* were 2.7, 16.5, and 1.9 times higher than those in sham control, respectively.

Next, the expression change in representative proteins was examined (Figure 2E), and the quantified results are depicted in Figure S6 (Supporting Information). The expression of iNOS, an N1 marker, peaked on day 1 post‐IRE and then waned off on day 7 post‐IRE. In comparison, the expression of Arg1, an N2 marker, dropped to a nadir on day 1 post‐IRE and then returned to baseline on day 7 post‐IRE. The expression of TGF‐*β*, p‐SMAD2, and three tumor proliferation markers (p‐PI3K, p‐p38, and p‐ERK1/2) exhibited a similar trend as that of Arg1. Interestingly, the expression of p‐PI3K and p‐ERK1/2 on day 7 post‐IRE was 2.8 and 1.7 times higher than those of sham control, indicating that although IRE temporarily suppressed tumor proliferation, the residual tumor cells recovered rapidly. These findings were in line with the change in the frequency of Ki67^+^ proliferating cells (Figure 2F), which was at the lowest level on day 1 post‐IRE with a gradual increase over time.

We then asked how the TAN phenotype shifted as the tumor recovered from IRE. TANs were isolated from Panc02 tumors on day 1 or day 7 post‐IRE with a purity above 90% (Figure S7, Supporting Information), and analyzed for mRNA expression of N2‐associated genes (Figure S8, Supporting Information). TANs were also isolated from sham control tumors to represent the N2 phenotype because of their extended exposure to the immunosuppressive cues in the tumor microenvironment.^[^ ^24^ ^]^ Thirteen out of the 14 tested genes had lower mRNA expression than sham control on day 1 post‐IRE, indicating that the freshly infiltrated neutrophils were less N2‐like. On day 7 post‐IRE, the mRNA expression of 11 genes bounced back toward the level of sham control, suggesting a shift toward the N2 phenotype. Notably, the mRNA level of *Arg1* on day 7 post‐IRE was 1.3 times higher than that in sham control. Collectively, it was established that the IRE‐induced infiltrating neutrophils gradually shifted toward the N2 phenotype as the tumor recovered; the tumor microenvironment also became more immunosuppressive.

A neutrophil depletion study was then conducted, via systemic injection of an *α*Ly6G antibody,^[^ ^24^ ^]^ to investigate its impact on the outcome of IRE + *α*PD1 treatment. As shown in Figure S9, Supporting Information, Panc02‐bearing mice with depleted neutrophils exhibited a 63% increase in median survival (79 d in the IRE + *α*PD1 + *α*Ly6G group vs 48.5 d in the IRE + *α*PD1 group, *p* < 0.05). This group also had a higher percentage of mice without visible tumors at the end of the study (Figure S9B, Supporting Information).

### 2.3 dMSN‐SB Enhanced Pancreatic Cancer Response to Combined IRE and *α*PD1 Therapy and Induced Long‐Term Antitumor Memory

**Figure 3:**
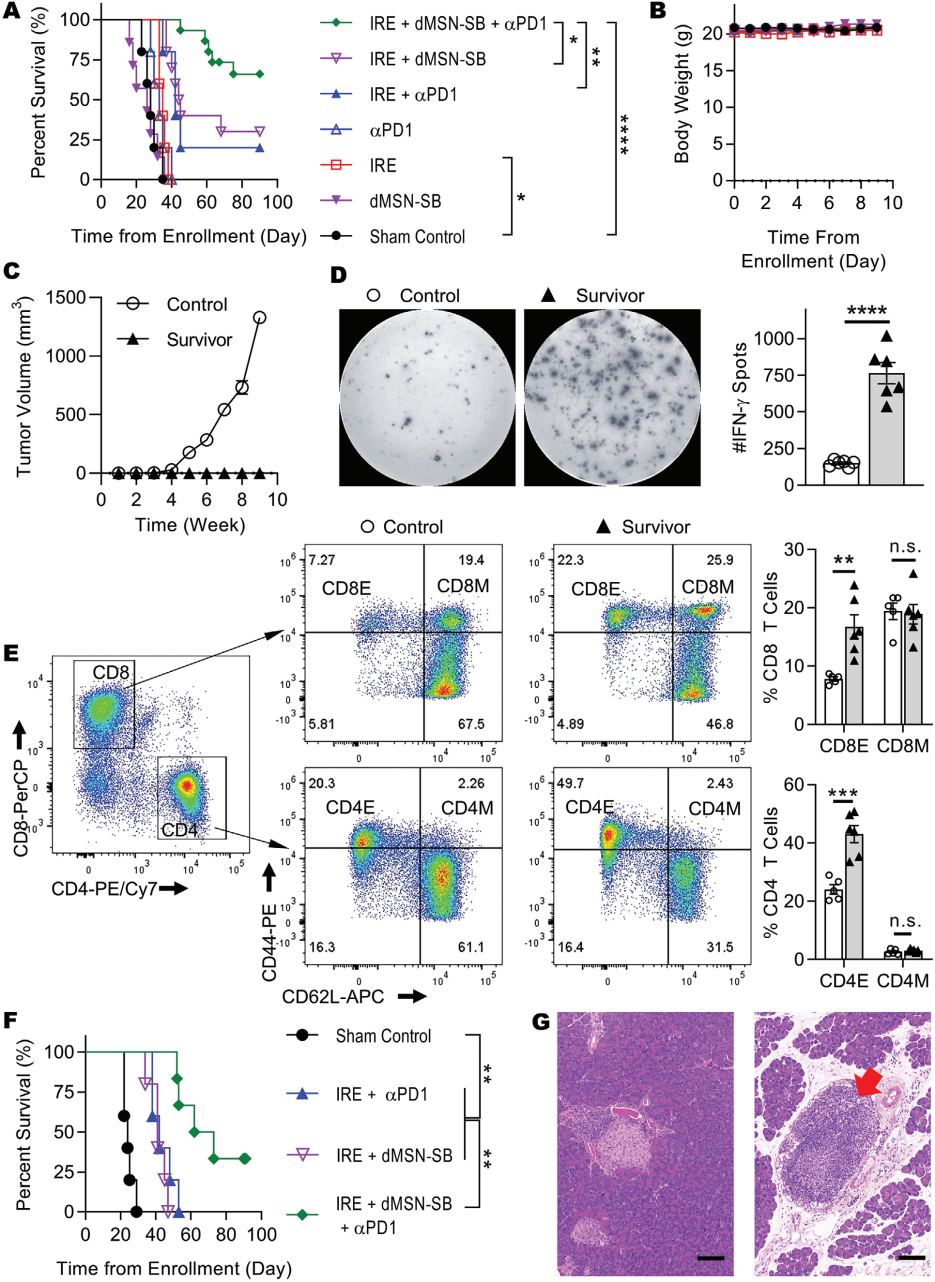
Antitumor efficacy in Panc02 models. A) Kaplan‐Meier survival curves and B) body weight of subcutaneous Panc02 model in groups of sham control (*n* = 5), IRE (*n* = 5), dMSN‐SB (*n* = 7), *α*PD1 (*n* = 5), IRE + *α*PD1 (*n* = 5), IRE + dMSN‐SB (*n* = 10), IRE + dMSN‐SB + *α*PD1 (*n* = 15). C) Growth curves of rechallenged Panc02 tumors in untreated control mice (*n* = 5) and long‐term survivors (*n* = 12). D) Representative images and quantification of IFN‐*γ* spots from the splenocytes of untreated control (*n* = 6) and long‐term survivors (*n* = 6). E) Representative flow cytometry plots and quantification of effector (CD44^+^CD62L^–^) and memory (CD44^+^CD62L^+^) T cells isolated from the spleens of untreated control (*n* = 5) and long‐term survivors (*n* = 6). CD4M, CD4 memory cells (CD4^+^CD44^+^CD62L^+^); CD4E, CD4 effector cells (CD4^+^CD44^+^CD62L^–^); CD8M, CD8 memory cells (CD8^+^CD44^+^CD62L^+^); CD8E, CD8 effector cells (CD8^+^CD44^+^CD62L^–^). F) Kaplan‐Meier survival curves of orthotopic Panc02 model in groups of sham control (*n* = 5), IRE + *α*PD1 (*n* = 5), IRE + dMSN‐SB (*n* = 5), IRE + dMSN‐SB + *α*PD1 (*n* = 6). G) Representative hematoxylin‐eosin staining of tumor‐free pancreas in orthotopic Panc02 model after treatment with IRE + dMSN‐SB + *α*PD1. Scale bar = 100 µm. Survival difference in (A) and (F) was evaluated using Log‐rank test. Data in (B–E) are presented as mean ± SEM in symbols or bar graphs overlaid with individual data points. Statistical difference was evaluated using unpaired Student's two‐tailed *t*‐test . *p < 0.05, **p < 0.01, ***p < 0.001, ****p < 0.0001, n.s. not significant.

The performance of dMSN‐SB was then evaluated in animal models. The nanoparticles were intratumorally injected right after IRE to maximize drug deposition within the tumor. Optical tracking of fluorescence‐labeled dMSN‐SB revealed that more than 70% of injected nanoparticles were retained inside the tumor on day 8 postinjection, and the leakage to other normal organs was minimal (Figure S10, Supporting Information). We first screened the survival of subcutaneous Panc02 model after different treatments (**Figure** **3**A). Monotherapy with IRE or *α*PD1 moderately improved the median survival from 28 d in sham control to 35 and 33 d, respectively, but neither treatment produced long‐term survival. Treatments with IRE + *α*PD1 or IRE + dMSN‐SB significantly improved median survival to 42 and 44.5 d respectively; while 20% of the mice treated with IRE + *α*PD1 and 30% of the mice treated with IRE + dMSN‐SB had no visible tumors at the end of the study. In comparison, 67% of the mice treated with triple therapy with IRE + dMSN‐SB + *α*PD1 had no visible tumors at the end of study, which was significantly higher than that in the groups of IRE + *α*PD1 or IRE + dMSN‐SB (Figure S11A, Supporting Information, *p* < 0.0001, *χ* ^2^ test). A similar trend of efficacy was observed in tumor growth curves. At 28 d after enrollment, mice treated with the triple treatment had the smallest tumors among all the groups (Figure S11B, Supporting Information). There was no significant decrease in body weight during the treatments (Figure 3B). We also compared free SB525334 with dMSN‐SB in their potency to enhance the efficacy of IRE + *α*‐PD1 (Figure S12, Supporting Information). SB525334 was administered via oral gavage at a dose of 10 mg kg^–1^ six times over 11 d, while dMSN‐SB was intratumorally injected at a dose of 10 mg kg^–1^ twice over two weeks. Panc02‐bearing mice in both IRE + dMSN‐SB + *α*PD1 and IRE + SB525334 + *α*PD1 groups had similar overall survival, while the IRE + dMSN‐SB + *α*PD1 group had a marginally higher percentage of mice without visible tumors (67% vs 54%, *p* = 0.06, *χ* ^2^ test). Therefore, it was demonstrated that dMSN‐SB could achieve similar therapeutic efficacy with less frequent dosing and lower total dosage.

Panc02‐bearing mice in the triple therapy group were further explored whether the treatment induced antitumor memory. The experiment timeline is shown in Figure S13, Supporting Information. Though the survivors and untreated tumor‐naïve mice were challenged by subcutaneous inoculation of Panc02 cells, only the tumor‐naïve mice experienced a robust tumor growth (Figure 3C). After the rechallenge study, splenocytes from the survivors were collected to estimate their secretion of interferon‐*γ* (IFN‐*γ*) in response to Panc02 cell lysate. ELISPOT assay showed that the splenocytes from the survivors produced 5 times more IFN‐*γ* spots than those of tumor‐naïve control (Figure 3D). The splenocytes were also stained with CD44 and CD62L to identify effector (CD44^+^CD62L^–^) and memory (CD44^+^CD62L^+^) T cells (Figure 3E). The representative gating strategy is presented in Figure S14, Supporting Information. Splenocytes from the survivors contained 1.8 times more effector CD8^+^ T cells and 2.1 times more effector CD4^+^ T cells than the control splenocytes. There was no significant difference in the frequencies of memory T cells.

We further evaluated the animal survival in groups of sham control, IRE + *α*PD1, IRE + dMSN‐SB, and the triple therapy using orthotopic Panc02 model (Figure 3F), because the pathological features of orthotopic pancreatic tumor models are more relevant to those of the human disease.^[^ ^25^ ^]^ Monotherapies were not included since they lacked efficacy in the subcutaneous model. Mice treated with the triple therapy had the longest median survival (67.5 d) among the four groups, and two out of the six mice had no palpable tumors at the end of the study, which was confirmed by histological examination of the pancreas (Figure 3G and Figure S15, Supporting Information). Interestingly, a tertiary lymphoid‐like structure was observed (red arrow), which has been reported to favor the outcome of immunotherapy.^[^ ^26^ ^]^ It should be noted that IRE has also been used in the clinical and preclinical treatment of liver,^[^ ^27^ ^]^ breast,^[^ ^28^ ^]^ renal cancers,^[^ ^29^ ^]^ etc. To test if our findings could extend to other tumor types, the treatment regimens were further evaluated in orthotopic 4T1 murine breast cancer model with a positive TGF‐*β* expression (Figure S5, Supporting Information). As shown in Figure S16, Supporting Information, mice treated with the triple therapy had the lengthiest median survival (33 d), and one out of the six treated mice had no visible tumor at the end of the study.

### 2.4 Analyses of Intratumoral Immune Cells

**Figure 4:**
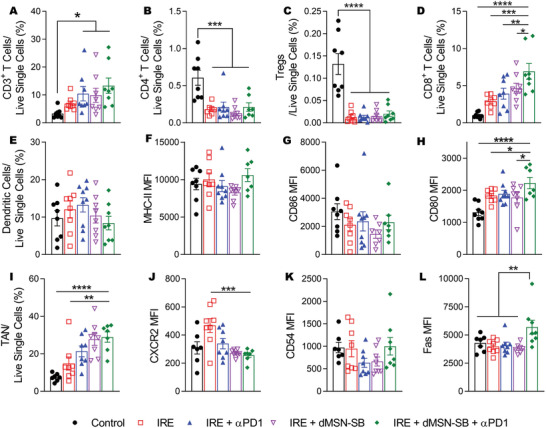
Analyses of intratumoral immune cells. A–D,E,I) Relative abundance of A) CD3^+^ T cells, B) CD4^+^ T cells, C) CD8^+^ T cells, D) Tregs, E) dendritic cells (DCs), and I) TANs in live single cells. Identification of each cell population are listed as follows: CD3^+^ T cells: CD45^+^CD3^+^, CD4^+^ T cells: CD45^+^CD3^+^CD4^+^; CD8^+^ T cells: CD45^+^CD3^+^CD8^+^ T cells, regulatory T (Tregs): CD45^+^CD3^+^CD4^+^CD25^+^Foxp3^+^; DC: CD45^+^CD11c^+^MHC‐II^+^; TAN: CD45^+^CD11b^+^Ly6G^+^. F–H) Mean fluorescence intensity (MFI) of F) MHC‐II , G) CD86, and H) CD80 in DCs. MFI of J) CXCR2, K) CD54, L) Fas in TANs. Data are presented as mean ± SEM of *n* = 8 independent biological samples in bar plots overlaid with individual data points. Significance of differences was determined using one‐way ANOVA followed by Tukey post‐hoc analysis. *p < 0.05, **p < 0.01, ***p < 0.001, ****p < 0.0001.

The immune microenvironment in the groups of sham control, IRE, IRE + *α*PD1, IRE + dMSN‐SB, and IRE + dMSN‐SB + *α*PD1 were then studied. Subcutaneous Panc02 tumors were collected on day 7 after mice enrollment and analyzed with flow cytometry. Treatment schedules and representative gating strategies are presented in Figures S17–S19, Supporting Information. Compared to sham control, the four groups involving IRE had 2 to 4 times more CD3^+^ T cells, 65% to 80% fewer CD4^+^ T cells, and remarkably 96% fewer regulatory T cells (Tregs) (**Figure** **4**A–C). The triple therapy group had the highest abundance of CD8^+^ T cells, which was 6.9, 2.4, 1.7, and 1.4 times higher than those of sham control, IRE, IRE + *α*PD1, and IRE + dMSN‐SB, respectively (Figure 4D). While there was no difference in dendritic cell abundance or the mean fluorescence intensity (MFI) of major histocompatibility complex‐II (MHC‐II) and CD86 (Figure 4E–G), the triple therapy group showed the highest MFI of CD80, a costimulatory molecule and surface marker for dendritic cell maturation (Figure 4H). All four groups involving IRE had more TANs than sham control (Figure 4I), which corroborated with our IHC results that IRE caused neutrophil infiltration (Figure 2B,C). Analyses of the surface markers on TANs showed that the MFI of CXCR2, an N2 marker, in the triple therapy and IRE + dMSN‐SB groups was only half that of the IRE group (*p* < 0.001, Figure 4J). While there was no significant difference in the MFI of CD54 (Figure 4K), an N1 marker; the MFI of Fas, another surface marker for N1 TAN, was 40% higher in the triple therapy group than in other groups (*p* < 0.01, Figure 4L). These results suggested that TGF‐*β* inhibition promoted TAN polarization toward the N1 phenotype.

### 2.5 Analyses of Intratumoral Cytokines and TAN Phenotypes

**Figure 5:**
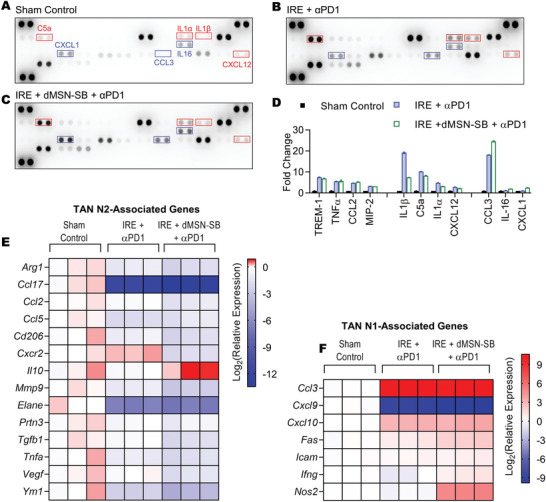
Analyses of intratumoral cytokines and TAN phenotype. Cytokine array analyses of A) subcutaneous Panc02 tumors from sham control, B) IRE + *α*PD1, C) IRE + dMSN‐SB + *α*PD1 groups. Exposure time = 600 s. D) Quantification of cytokines whose expression was significantly altered after treatment with IRE + *α*PD1 or the triple therapy. Cytokines with higher expression in the IRE + *α*PD1 group were indicated using red boxes; those with higher expression in the triple therapy group were marked with blue boxes. Two dots were probed for each cytokine. Data are presented as mean ± SEM in bar graphs. Significant difference was evaluated using one‐way analysis of variance for multiple comparisons followed by Tukey post hoc analysis. Relative expression of E) N2‐ and F) N1‐ associated genes in TANs isolated from subcutaneous Panc02 tumors on day 7 after enrollment. Data are shown as heat map with a color scale. Each square represents an individual independent data point. Three replicates were included in each group.

Next, the expression of 40 intratumoral cytokines was compared among the sham control, IRE + *α*PD1, and the triple therapy groups, on day 7 after enrollment of mice with subcutaneous Panc02 tumors (**Figure** **5**A–D). Eleven cytokines were significantly upregulated after treatment with either IRE + *α*PD1 or the triple therapy (Figure 5D). Gene Ontology analysis revealed that the top five enriched biological processes were leukocyte migration, leukocyte chemotaxis, cytokine‐mediated signaling, cell chemotaxis, and inflammatory response (Table S1, Supporting Information), suggesting that tumors in the IRE + *α*PD1 or triple therapy group had a proinflammatory microenvironment with robust immune activities. Compared to IRE + *α*PD1, the triple therapy group had lower expression in IL‐1*β*, C5a, IL‐1*α*, and CXCL12 (marked with red boxes) but higher expression in CCL3, IL‐16, and CXCL1 (marked with blue boxes).

TANs were then isolated from subcutaneous Panc02 tumors of the sham control, IRE + *α*PD1, and the triple therapy groups on day 7 after mice enrollment, and analyzed for their expressions of N2‐ and N1‐associated genes (Figure 5E,F).^[^ ^16^ ^]^ The mRNA expression of CCL3 in both IRE + *α*PD1 and triple therapy groups was 1000 times higher than that in sham control, which was in accordance with the findings of cytokine array (Figure 5D). Compared to the IRE + *α*PD1 group, the triple therapy group exhibited lower mRNA expression in 12 out of 14 N2‐associated genes, and higher mRNA expression in five out of six N1‐associated genes. Notably, the mRNA levels of *Fas* and *Nos2*, two N1‐associated genes, were 2.2 and 41.4 times higher in the triple therapy group than in the IRE + *α*PD1 group. The difference in *Fas* mRNA expression also agreed with the flowcytometry data where the MFI of Fas in the triple therapy was 40% higher than that in the IRE + *α*PD1 group (Figure 4L). The combined results demonstrated that the intratumoral inhibition of TGF‐*β* by dMSN‐SB successfully shifted TANs toward the N1 phenotype.

### 2.6 Treatment‐Induced Changes in the Stromal Components

**Figure 6:**
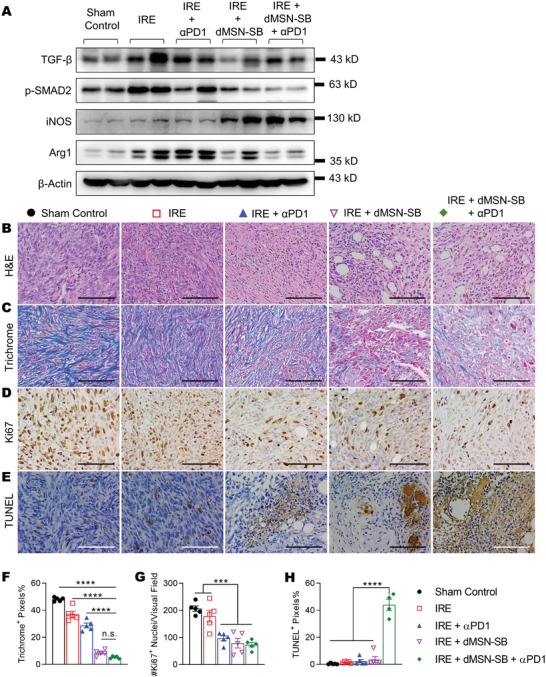
Analyses of tumor stroma. A) Immunoblotting of TGF‐*β*, p‐SMAD2, iNOS, and arginase 1. Two independent Panc02 tumors were included in each group. Representative microscopic images of B) H&E, C,F) trichrome, D,G) Ki67, E,H)TUNEL IHC staining and respective quantification. Subcutaneous tumors were collected on day 7 after enrollment. Scale bar = 100 µm. Five 400× visual fields were imaged at random for quantification. Data in (B–H) are presented as mean ± SEM in bar graphs overlaid with individual data points. Significance was determined using one‐way ANOVA followed by Tukey post hoc analysis. ***p < 0.001, ****p < 0.0001, n.s. = not significant.

Stromal components in sham control, IRE, IRE + *α*PD1, IRE + dMSN‐SB, and the triple therapy were subsequently studied on day 7 after enrollment (**Figure** **6**). The levels of p‐SMAD2 in IRE + dMSN‐SB and the triple therapy were lower than those in the other groups, confirming the successful inhibition of TGF‐*β* by dMSN‐SB (Figure 6A and Figure S20, Supporting Information). The triple therapy groups had higher iNOS but lower Arg1 levels than the IRE + *α*PD1 group, which was in agreement with the mRNA expression levels depicted in Figure 5E,F and thereby indicated a shift of TAN toward the N1 phenotype.

The residual tumor regions were also examined with H&E and IHC staining. Pathological analyses (Figure 6B) showed that tumors in sham control were composed of poorly differentiated cancer cells with enlarged and hyperchromatic nucleus, multiple nucleoli, and pathological mitosis. The cancer cells were diffusely distributed without glandular or solid nests, and occasional ulceration was visible. In contrast, tumors from the triple therapy group exhibited extensive necrosis ranging from focal spots to throughout the whole tumor. Acute inflammation with hyperemia and neutrophil infiltration was observed at the borders between the necrotic and viable regions. Increased frequency of glandular nests was found within the treated tumor regions. The IRE + dMSN‐SB and triple therapy groups had similar collagen content (Trichrome staining, Figure 6C,F), which was significantly lower than those in the other groups. The reduction in collagen content was consistent with the antifibrosis activities of TGF‐*β* inhibitors. The frequency of Ki67^+^ proliferating cells was similar in the residual tumor regions of the IRE + *α*PD1, IRE + dMSN‐SB, and the triple therapy groups, and these were significantly lower than those in the sham control or IRE groups (Figure 6D,G). The triple therapy group exhibited significantly more apoptosis (TUNEL staining, Figure 6E,H) than the other groups. Taken together, we have shown that the triple therapy reshaped the tumor microenvironment in favor of immunotherapy, and successfully suppressed tumor proliferation.

### 2.7 Toxicity Profiles of IRE + dMSN‐SB + *α*PD1 Treatment

**Figure 7:**
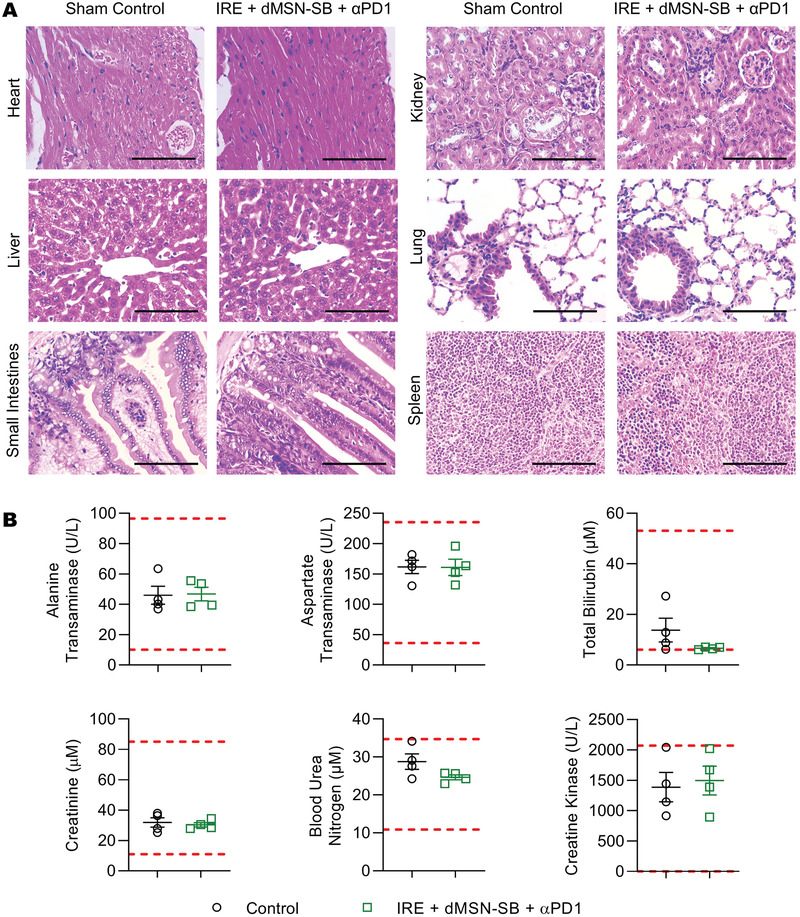
Toxicity analyses of IRE + dMSN‐SB + *α*PD1 treatment. A) Representative H&E staining of major organs at 400× magnification. Scale bar = 100 µm. B) Blood chemistry analyses of liver, kidney, and heart toxicity. Data are presented as mean ± SEM of *n* = 4 in scattered dot plots. The upper and lower limits of normal ranges are marked as red dashed lines.

We then investigated whether the triple therapy induced toxic reactions in major organs in the orthotopic Panc02 model. H&E staining of heart, liver, small intestines, kidney, lung, and spleen revealed no pathological alterations (**Figure** **7**A). Analysis of the blood chemistry showed that indexes for the functions of the liver (alanine transaminase, aspartate transaminase, and total bilirubin), the kidney (creatine and blood urea nitrogen), and the heart (creatine kinase) were all within the normal ranges (Figure 7B).

## 3 Discussion

In this study, we prepared a GSH‐responsive degradable mesoporous silica nanoformulation (dMSN‐SB) that inhibited TGF‐*β* signaling within the tumor microenvironment, promoted TAN polarization toward the anti‐tumor N1 phenotype, enhanced pancreatic cancer response to combined IRE and *α*PD1 therapy, and induced long‐term antitumor memory.

IRE is a novel local ablative technique with several advantages over conventional ablative modalities. First, IRE kills tumor cells by permanent disruption of cell membrane rather than the heating effect.^[^ ^4^ ^]^ Its efficacy is not compromised by large blood vessels that can dissipate heat and render thermal ablation ineffective. Second, IRE preserves the extracellular matrix of vasculatures and shows better safety profiles in the vicinity of vital structures such as blood vessels, bile ducts, and the urinary tract.^[^ ^30^ ^]^ However, the cytotoxicity of IRE depends on the strength of electric pulses, which decreases as the distance between the tumor cells and electrodes increases. The intratumoral heterogeneity may also form regions of low pulse strength where tumor cells can survive.[^31^] Indeed, local tumor recurrence post‐IRE is often attributed to incomplete ablation. Although adjuvant chemotherapies can be used perioperatively as a preventive approach,^[^ ^32^ ^]^ the associated toxicity may further exacerbate patient health. In addition to direct tumor killing, IRE can boost antitumor immunity by releasing damage‐associated molecular patterns and tumor‐associated antigens, a process known as in situ vaccination.^[^ ^28^ ^]^ It also transiently alleviates immunosuppression and enhances the efficacy of other immunotherapy modalities.^[^ ^4^ ^]^ IRE‐induced modulation of the immune microenvironment was also observed in this study. For example, all treatment groups involving IRE had more intratumoral CD3^+^ and CD8^+^ T cells (Figure 4A,D), which was consistent with the elevated levels of CCL3 (Figure 5D), a T cell‐recruiting cytokine.^[^ ^33^ ^]^ Accordingly, the depletion of Tregs in IRE‐involved groups (Figure 4C) was in line with the decrease in the mRNA transcription of CCL17 (Figures 2D and 5E), which is a strong chemoattractant for Tregs.^[^ ^34^ ^]^

We observed a rapid infiltration of neutrophils into the pancreatic tumor after IRE via IHC staining (Figure 2B,C) and flowcytometry (Figure 4I), which was consistent with the acute inflammation following IRE‐induced tumor necrosis (Figure 2A). Neutrophils are the most abundant subpopulation of circulating leukocytes, and play a complex and fluidic role during tumor progression.^[^ ^13^ ^]^ TANs can kill cancer cells and inhibit metastasis by releasing reactive oxygen species (ROS) and potentiate antitumor T cell response after TGF‐*β* inhibition.^[^ ^35^ ^]^ Conversely, TANs may also favor genetic instability by releasing ROS, secrete cytokines to promote tumor proliferation, invasion, and angiogenesis, and exert immunosuppression by expression of arginase.^[^ ^35^ ^]^ We monitored the longitudinal changes in the N2‐associated genes in tumor lysate and isolated TANs (Figure 2 and Figure S8, Supporting Information), and demonstrated that not only the infiltrating neutrophils polarized toward the protumor N2 phenotype after IRE, but also the tumor microenvironment became more immunosuppressive. This finding was in line with the pancreatic cancer expression of TGF‐*β* in both patient samples^[^ ^23a^ ^]^ and animal models (Figure S5, Supporting Information). Notably, the abundance of TANs was higher than that of each T cell subpopulations in all treatment groups (Figure S21, Supporting Information), suggesting that TANs may play critical roles in the therapeutic outcome of IRE. To the best of our knowledge, this is the first evidence suggesting that TANs may be responsible for the unsatisfactory outcome of IRE‐based immunotherapies. Our finding corroborated with a recent study showing that suppression of TANs by lorlatinib attenuates pancreatic cancer growth and enhances immune checkpoint blockade.^[^ ^36^ ^]^

TGF‐*β* signaling plays an important role in tumor initiation as well as progression, exerts systemic immunosuppression, and inhibits host immunosurveillance.^[^ ^37^ ^]^ In advanced PDAC, inhibition of TGF‐*β* alleviated stromal fibrosis, restored function to intratumoral vasculature, mitigated hypoxia, and enhanced the efficacy of chemotherapy and immunotherapy.^[^ ^38^ ^]^ On the other hand, TGF‐*β* signaling and the cytokines of TGF‐*β* family are also involved in many cellular, pathological, and physiological processes, such that the inappropriate disruption of TGF‐*β* signaling is associated with numerous diseases. For example, impaired TGF‐*β*/SMAD3 signaling has been reported to promote aortic aneurysm formation and rupture.^[^ ^39^ ^]^ Disruption of TGF‐*β* signaling is linked to low CD39 expression of regulatory T cells and leads to drug resistance of rheumatoid arthritis.^[^ ^40^ ^]^ TGF‐*β* signaling is also critical for wound healing process.^[^ ^41^ ^]^ Although toxicological studies on SB525334 have been scarce, the adversary effects of galunisertib, another TGF*β*1 receptor inhibitor, have been reported in both clinical and pre‐clinical studies.^[^ ^17^ ^]^ Importantly, chronic oral administration of galunisertib results in multiple target organ toxicities involving the cardiovascular, gastrointestinal, immune, bone/cartilage, reproductive, and renal systems.^[^ ^42^ ^]^ In our studies, we observed abnormally high levels of alanine transaminase, aspartate transaminase, blood urea nitrogen, and creatine kinase in mice treated with IRE + SB525334 (Figure S22A, Supporting Information). Regions of hydropic degeneration, fatty degeneration, and myolysis were also observed in the heart section of mouse treated with IRE + SB525334 (Figure S22B, Supporting Information). Taken together, we concluded that systemic oral administration of SB525334 was not the optimal approach to TGF‐*β* inhibition within the tumor microenvironment.

We developed dMSN‐SB for the local release of TGF‐*β* inhibitor. The in vivo modulation of TAN polarization was evident by the decrease in CXCR2 (N2 marker) and the increase in Fas (N1 marker) by flowcytometry (Figure 4J,L). These results were consistent with the comparison of mRNA expression of N1‐ and N2‐associated genes between the IRE + *α*PD1 and the triple therapy (Figure 5E,F). Tumor lysate of the triple therapy also exhibited 5.6 time higher expression of iNOS (N1 marker) and 33% lower expression of Arg1 (N2 marker) than those of the IRE + *α*PD1 group, respectively (Figure 6A). Collectively, we have demonstrated that dMSN‐SB indeed induced TAN polarization toward the anti‐tumor N1 phenotype. Given the elevated abundance of TANs in post‐IRE tumors (Figure 5I), it was likely the N1‐polarized TANs enhanced the antitumor efficacy of combined IRE and *α*PD1 therapy. In the meantime, SB525334 was reported to have mild cytotoxicity to cancer cells, and showed synergistic effects with gemcitabine and doxorubicin.^[^ ^43^ ^]^ To investigate the possibility that the cytotoxicity of SB525334 enhanced the efficacy of IRE, we examined the in vitro cytotoxicity of reversible electroporation and SB525334. This setup was to mimic the tumor regions where SB525334 was present but the pulse intensity was below the threshold of IRE. As shown in Figure S23, Supporting Information, SB525334 did not enhance cell killing by reversible electroporation. Therefore, the major role of dMSN‐SB in the enhancement of IRE + *α*PD1 should be attributed to its inhibition of TGF‐*β* signaling and subsequent modulation of TANs.

IL‐1*α* and IL‐*β* belong to the IL‐1 family and are required for activation of innate immune cells.^[^ ^44^ ^]^ But a persistent upregulation of IL‐1 could promote fibrosis and macrophage‐mediated immunosuppression.^[^ ^45^ ^]^ C5a is a complement protein and its upregulation causes vasodilation and increases vascular permeability, both of which facilitate tumor infiltration by immune cells.^[^ ^46^ ^]^ Yet, a persistent activation of C5a could promote immunosuppression.^[^ ^47^ ^]^ CXCL12 is known to cause immune resistance in pancreatic cancer and promote cancer progression.^[^ ^48^ ^]^ CCL3 regulates the recruitment of neutrophils, dendritic cells, and CD8^+^ T cells,^[^ ^49^ ^]^ while CXCL1 is a known chemoattractant for neutrophils.^[^ ^35^ ^]^ IL‐16 is primarily associated with regulation of T cell growth, and recruitment of CD4^+^ cells during inflammation.^[^ ^50^ ^]^ Taken together, we have shown that the triple therapy reshaped the cytokine milieu in favor of long‐term antitumor immunity (Figure 5A–D). Our study has several limitations. First, the exact interaction between TANs and tumor‐infiltrating T cells remains to be elucidated. The intratumoral release profile of dMSN‐SB can be further tuned to achieve an optimal and prolonged inhibition of TGF‐*β* signaling.

## 4 Conclusion

Although IRE is a clinically approved ablative technique for the treatment of advanced PDAC, tumor recurrence post‐IRE remains an obstacle to overcome. We herein established that IRE induces a rapid infiltration of neutrophils into pancreatic tumors. The infiltrating neutrophils then polarize toward the protumor N2 phenotype and can potentially compromise the efficacy of IRE‐based immunotherapy. We further designed a GSH‐responsive mesoporous silica nano‐formulation (dMSN‐SB) that inhibits intratumoral TGF‐*β* signaling, promotes TAN polarization toward the anti‐tumor N1 phenotypes, and subsequently enhances pancreatic cancer response to combined IRE and *α*PD1 therapy. In summary, our results suggest that TAN modulation is a promising and safe strategy to further enhance response of pancreatic cancer to IRE‐based immunotherapy.

## 5 Experimental Section

### Preparation of Degradable Mesoporous Silica Nanoparticles Loaded with SB525334

All chemicals were purchased and used as received from Aladdin Biochemical Technology (Shanghai, China) or Sigma‐Aldrich (MO, United States). GSH‐responsive degradable mesoporous silica nanoparticles (dMSN) were synthesized as previously described with some modification.^[^ ^21^ ^]^ Briefly, cetyltrimethylammonium chloride (CTAC, 0.35 g), Pluronic F‐127 (0.10 g), and triethylamine (0.8 mL) were dissolved in ultrapure water (100 mL) and stirred at 80 °C. A mixture of tetraethyl orthosilicate and bis(triethoxysilylpropyl)disulfide (1 mL each) was added dropwise. One hour later, 3‐(trihydroxysilyl)propyl methylphosphonate monosodium salt solution (42% by weight in water, 0.5 mL) was added, and the mixture was stirred at 80 °C for additional 3 h to form dMSN. CTAC and F‐127 were removed by refluxing in an ethanolic solution of hydrochloric acid overnight. The nanoparticles were then washed with water and collected by centrifugation. TGF‐*β*1 receptor 1 inhibitor, SB525334, was obtained from Target Molecule (Shanghai, China) and loaded in dMSN nanoparticles via adsorption method. Briefly, dMSN (1 mg) was dispersed in water (100 µL), followed by adding a solution of SB525334 (300 µg) in ethanol (20 µL). The mixture was agitated in dark at room temperature overnight, and centrifuged at 12 000 *g* for 10 min to remove the unencapsulated drug. The pellet (dMSN‐SB) was then redispersed in water for further use.

### Physiochemical Characterization of dMSN‐SB

Hydrodynamic diameter of particles was measured using dynamic light scattering on a ZetaPlus particle analyzer (Brookhaven Instrument, NY, USA). Particle morphology was examined on a JEOL JEM 2100F transmission electron microscope (TEM, JEOL USA, MA, USA) following established protocols.^[^ ^20a^ ^]^ To determine the efficiency of drug loading, the mixture of dMSN and SB525334 was centrifuged at 12 000 *g* for 10 min, the supernatants were collected and the absorbance at 370 nm read on a NanoDrop 2000 ultraviolet–visible (UV–vis) spectrometer (Thermo Fisher Scientific, Shanghai, China). Loading efficiency (%) was calculated as (total—unencapsulated SB525334)/total SB525334 × 100%. Drug release was studied by immersing dMSN‐SB‐filled dialysis bags (molecular weight cutoff = 14 000 Dalton) in phosphate‐buffered saline (PBS) at pH 7.4 or sodium acetate buffer at pH 5.2 supplemented with 20 × 10^‐3^ m GSH, and incubating them in a 37 °C water bath with constant shaking. Tween‐20 was added into the release media to facilitate cross‐membrane transport of the released payload. At predetermined time points, aliquots of dMSN‐SB suspension inside the dialysis bags were collected, mixed with an equal volume of ammonia, and centrifuged. The pellets were redispersed in dimethyl sulfoxide, their absorbance at 370 nm was recorded as a measured of unreleased SB525334.

### Cell Lines and Animal Models

All animal studies were approved by the Institutional Animal Care and Use Committee of Huazhong University of Science and Technology and carried out in accordance with institutional guidelines in specific pathogen‐free facilities. Panc‐1 human pancreatic cancer cells, HL‐60 human promyeloid cells, Panc02 murine pancreatic cancer cells, and 4T1 murine breast cancer cells were obtained from Cell Resource Center, Peking Union Medical College, the headquarter of National Infrastructure of Cell Line Resources. All cell lines were authenticated by short tandem repeat (STR) profiling and routinely tested for mycoplasma contamination.

The subcutaneous Panc02 tumor model was established by inoculating Panc02 cells (4 × 10^6^) at the lower left back of 6 week old C57BL/6 mice (SPF Biotechnology, Beijing, China). The orthotopic Panc02 model was established by intrapancreatic engraftment of tumor pieces. Mice were anesthetized with 2% isoflurane, then a small incision was made at the left abdomen to expose the spleen and pancreas. Freshly isolated subcutaneous Panc02 tumor was cut into 2 mm^3^ pieces and inserted into a small pocket of the pancreas. The pocket was closed with absorbable sutures, followed by closing the incision with absorbable sutures and Vetbond Tissue Adhesive (3M Science, Wuhan, China). The Orthotopic 4T1 murine breast cancer model was established by inoculating 4T1 cells (2 × 10^6^) at the mammary fat pad of female BALB/c mice.

### Electroporation and In Vivo Antitumor Efficacy

Electroporation was performed using a two‐needle array electrode with a 5 mm gap made of medical grade stainless steel (BTX item #45‐0168, BTX Harvard Apparatus, MA, USA). Electric pulses were generated using ECM 830 Square Wave Electroporation System (BTX Harvard Apparatus) with the following parameters: voltage = 1200 V, pulse duration = 100 µs, pulse repetition frequency = 1 Hz, number of repetition pulses = 99. For subcutaneous Panc02 tumors, the two‐needle array was inserted through the skin into the center of tumor nodule along the long axis. For orthotopic Panc02 tumors, mice were anesthetized and a 5 mm incision was made at the left abdomen to expose the tumor nodule. Next, the two‐needle array was inserted such that it fully penetrated the tumor nodule to maximize the effect of electroporation (Figure S24, Supporting Information).^[^ ^6^ ^]^

Tumor‐bearing mice were enrolled once the tumor size reached 7 mm in one dimension measured by a caliper. They were randomly assigned to the following groups: sham control, IRE, *α*PD1, dMSN‐SB, IRE + dMSN‐SB, IRE + *α*PD1, or IRE + dMSN‐SB + *α*PD1. For subcutaneous model, dMSN‐SB was suspended in ultrapure water at 20 mg mL^–1^ (in SB525334 equivalent) and intratumorally injected at 10 µL per injection, one injection per week for 2 weeks. For the orthotopic Panc02 model, the tumor growth was monitored by palpation under anesthesia. Due to the thin skin and muscle of the mouse, one could identify tumors of about 7 mm in length in 90% of the mice, while animals with larger tumors were excluded. After IRE treatment of the orthotopic Panc02 tumor, 20 µL dMSN‐SB was intratumorally injected once only right after IRE. The *α*PD‐1 antibody (RMP1‐14 clone, BE0146, BioXCell, NH, USA) was intraperitoneally injected at 100 µg per injection, starting from 30 min after IRE, followed by another injection every 48 h up till six total doses.^[^ ^6^ ^]^ For neutrophil depletion, *α*Ly6G antibody (clone 1A8, BE0075‐1, BioXCell) was intraperitoneally administered at 100 µg per injection, starting from day 1 before IRE, and thereafter one injection was administered every 48 h up to six total doses.

### In Vitro Polarization of Murine Bone Marrow‐Derived Neutrophils

Naïve neutrophils were isolated via density gradient centrifugation using a murine bone‐marrow neutrophil isolation kit (TBD2013NM, TBD Science, Tianjin, China), and cultured at 37 °C in RPMI 1640 medium supplemented with 10% fetal bovine serum (FBS) and pan‐caspase inhibitor QVD‐OPh (3 × 10^‐6^ m, Target Molecule, Shanghai, China). Neutrophil polarization was conducted by adding an N2 cocktail containing l‐lactate (25 × 10^‐3^ m), adenosine (10 × 10^‐6^ m), murine TGF‐*β* (20 ng mL^–1^), murine interleukin‐10 (10 ng mL^–1^), prostaglandin E2 (20 ng mL^–1^), and murine granulocyte colony‐stimulating factor (100 ng mL^–1^) to the culture medium.^[^ ^22^ ^]^ The pH of the N2‐polarizing medium was adjusted to 6.7 using hydrochloric acid. TGF‐*β* inhibition was achieved by adding SB525334 (10 × 10^‐6^ m) or dMSN‐SB (50 × 10^‐6^ m). Cells were cultured for 24 h before analyses.

### Quantitative RT‐PCR

Total RNA was isolated using a MicroElute Total RNA kit (R6831, Omega Bio‐Tek, Guangzhou, China), and transcribed to cDNA using HiScript II Q RT SuperMix (Vazyme, Nanjing, China). Real‐time PCR was performed using ChamQ SYBR qPCR Master Mix (Vazyme) with the primers listed in Table S2, Supporting Information, on a Bio‐Rad CFX96 RT‐PCR system (Bio‐Rad, CA, USA). Relative gene expression was calculated using the 2^–ΔΔ^ *^Ct^* method and normalized to *18S* endogenous control.

### Tumor Digestion and Flowcytometry Analyses of Immune Cells

Weighted tumors were cut into pieces and digested in a mixture comprising collagenase IV (1 mg mL^–1^), hyaluronidase (0.4 mg mL^–1^), DNase I (0.1 mg mL^–1^), and bovine serum albumin (10 mg mL^–1^) in RPMI‐1640 medium at 37 °C under constant shaking for 30 min. Debris was removed by filtration through a 40 µm mesh. After lysing red blood cells, the cell mixture was washed and resuspended in PBS supplemented with 2% FBS for further analyses.

Spleens were minced, filtered through 40 µm meshes, and subjected to red blood cell lysis to obtain suspension of splenocytes. The splenocytes or digested tumors were stained with Zombie NIRTM Fixable Viability Kit (Biolegend, USA), blocked with antimouse CD16/32, and then stained with the antibodies listed in Table S3, Supporting Information. Intracellular staining was performed after fixation and permeabilization with a Biolegend True‐Nuclear Transcription Buffer Set. Samples were analyzed on a BD FACSVerse 3L8C cytometer (San Jose, CA, USA). Data were processed with FlowJo software V10 (BD Biosciences, San Jose, CA, USA).

### ELISPOT Analyses of Interferon‐*γ* Secreting Splenic Cells

Splenic cell‐secreted interferon‐*γ* was analyzed using an ELISPOT assay kit (3321‐4HST‐2, MabTech, Sweden). Murine dendritic cells were generated from bone marrow after 8 d of culture in RPMI1640 medium supplemented with 10% heat‐inactivated FBS, granulocyte‐macrophage colony‐stimulating factor (20 ng mL^–1^), and *β*‐mercaptoethanol (50 × 10^‐6^ m).^[^ ^6^ ^]^ Panc02 cell lysate was prepared by 10 cycles of freeze‐and‐thaw. Dendritic cells were pulsed with the lysate for 3 h and cocultured with splenocytes of untreated or long‐term surviving mice overnight at 37 °C. Formed IFN‐*γ* spots were then imaged and counted.

### Magnetic‐Activated Cell Sorting for Tumor‐Associated Neutrophils

A single‐cell suspension of the digested tumor was blocked with 5% FBS in tris buffered saline supplemented with 0.1% tween‐20 (TBST) and sequentially incubated with PE‐conjugated *α*Ly‐6G antibody (Biolegend) and anti‐PE microbeads (Miltenyi Biotec, Shanghai, China). Neutrophils were positively selected using a MACS LS column (Miltenyi Biotec).

### Western Blotting and Cytokine Array

Cell or tumor lysates were fractioned on tris‐glycine gels and transferred to polyvinylidene fluoride membranes (Millipore, Billerica, MA, USA). The membranes were blocked with 5% nonfat milk in TBST, blotted with primary antibodies (Table S4, Supporting Information) and horseradish peroxidase‐conjugated secondary antibodies, incubated with enhanced chemiluminescence substrate, and scanned on a Tanon 5200 Chem‐Image system (Tanon Science & Technology, Shanghai, China). Mouse cytokine array (Catalogue# ARY006, R&D Systems, MN, USA) was incubated with tumor lysate per manufacturer's instructions and imaged on a Tanon 5200 Chem‐Image system. Layout of the array is presented in Table S5, Supporting Information. Signal intensity of each target dot was integrated using Image J software.

### Immunohistochemical Staining

Paraffin‐embedded formalin‐fixed tissue sections were rehydrated and heated in 10 × 10^‐3^ m citrate buffer (pH 6.0) at 95 °C for antigen retrieval. The sections were then blocked with 10% goat serum in TBST and incubated with primary antibodies (Table S6, Supporting Information), biotinylated antirabbit IgG, and streptavidin‐conjugated horseradish peroxidase, respectively. Positive staining was detected via a 3,3'‐diaminobenzidine (DAB) reaction. The sections were then counterstained with hematoxylin and imaged on a bright‐filed microscope.

### Statistics

Details of data presentation, sample size (*n*), statistical analysis, and significance of differences are given in the figure captions. Differences between data sets were evaluated using unpaired Student's two‐tailed *t*‐test for the comparison of two data sets, and one‐way analysis of variance for multiple comparisons followed by Tukey post hoc analysis for comparison of three or more data sets. Log‐rank test was used for Kaplan‐Meier survival analysis. Difference in the percentage of mice without visible tumor was examined using *χ* ^2^ test. A *p*‐value less than 0.05 was considered statistically significant. Statistical analysis of data was performed using Prism GraphPad 9 (GraphPad Software Inc., San Diego, CA, USA).

## Conflict of Interest

The authors declare no conflict of interest.

## Author Contributions

H.P. and J.S. contributed equally to this work. H.P., J.S., X.L., X.Z., J.Z., X.X., T.H., H.X., and J.Z. performed the experiments. S.S., C.L., P.L., H.W., and J.Z. conceptualized the study. J.Z. drafted the manuscript.

## Supporting information

Supporting Information

## Data Availability

The data that support the findings of this study are available from the corresponding author upon reasonable request.
