# Supplementary material for: Local Release of TGF‐β Inhibitor Modulates Tumor‐Associated Neutrophils and Enhances Pancreatic Cancer Response to Combined Irreversible Electroporation and Immunotherapy
Source: Adv Sci (Weinh). 2022 Feb 7;9(10):2105240. doi: 10.1002/advs.202105240 (PMC8981446; doi:10.1002/advs.202105240)
Supplement: Supplementary file 1 — Supporting Information [file ADVS-9-2105240-s001.pdf]

ADVANCED SCIENCE
Open Access

Supporting Information

for *Adv. Sci.*, DOI 10.1002/advs.202105240

Local Release of TGF-β Inhibitor Modulates Tumor-Associated Neutrophils and Enhances Pancreatic Cancer Response to Combined Irreversible Electroporation and Immunotherapy

Huiming Peng, Jian Shen, Xin Long, Xiaoqi Zhou, Jiaqi Zhang, Xina Xu, Teng Huang, Hui Xu, Shuguo Sun, Chun Li, Ping Lei, Heshui Wu* and Jun Zhao**

ADVANCED SCIENCE
Open Access

Supporting Information

for *Adv. Sci.,* DOI: 10.1002/advs.202105240

Local Release of TGF-β Inhibitor Modulates Tumor-Associated Neutrophils and Enhances Pancreatic Cancer Response to Combined Irreversible Electroporation and Immunotherapy

Huiming Peng, Jian Shen, Xin Long, Xiaoqi Zhou, Jiaqi Zhang, Xina Xu, Teng Huang, Hui Xu, Shuguo Sun, Chun Li, Ping Lei, Heshui Wu*, and Jun Zhao**

Supporting Information for

Local Release of TGF-β Inhibitor Modulates Tumor-Associated Neutrophils and Enhances Pancreatic Cancer Response to Combined Irreversible Electroporation and Immunotherapy

Huiming Peng, Jian Shen, Xin Long, Xiaoqi Zhou, Jiaqi Zhang, Xina Xu, Teng Huang, Hui Xu, Shuguo Sun, Chun Li, Ping Lei, Heshui Wu*, Jun Zhao**

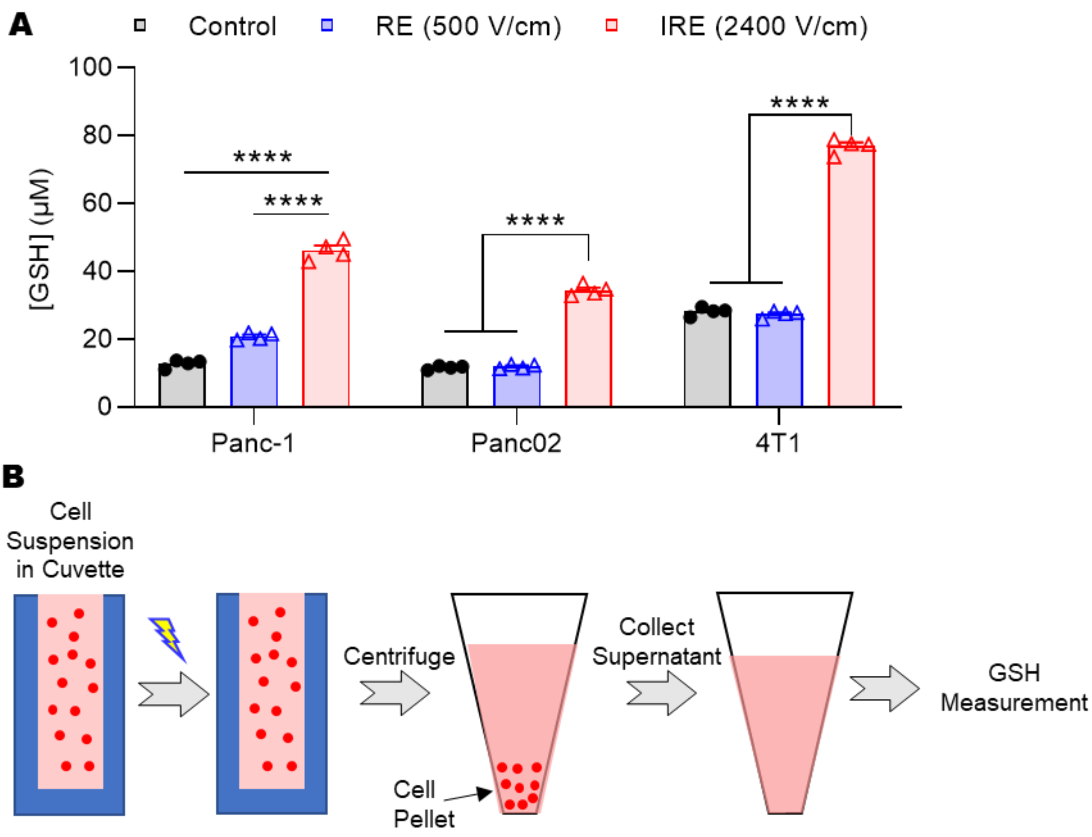

Figure S1. IRE induced release of glutathione (GSH). A) GSH concentration in cell supernatant after IRE treatment of Panc-1 human pancreatic cancer cells, Pan02 murine pancreatic cancer cells, and 4T1 murine breast cancer cells. B) Schemes of experiment flow. Cells were suspended in phosphate buffered saline (PBS) at 2×10^6/mL, and subjected to electroporation at 0 (control), 500 V/cm (reversible electroporation, RE), or 2400 V/cm (IRE). Other parameters for electroporation: pulse duration = 100 μsecond, pulse interval = 1 second, pulse number = 20. The supernatant of cell suspension was collected, GSH concentration measured with Ellman's reagent. ***p < 0.0001. Data are presented as mean ± SEM of $n = 4$ in bar graphs overlaid with individual data points. Significance was determined using one-way ANOVA followed by Tukey post hoc analysis. * p < 0.05, ** p < 0.01, n.s. = not significant

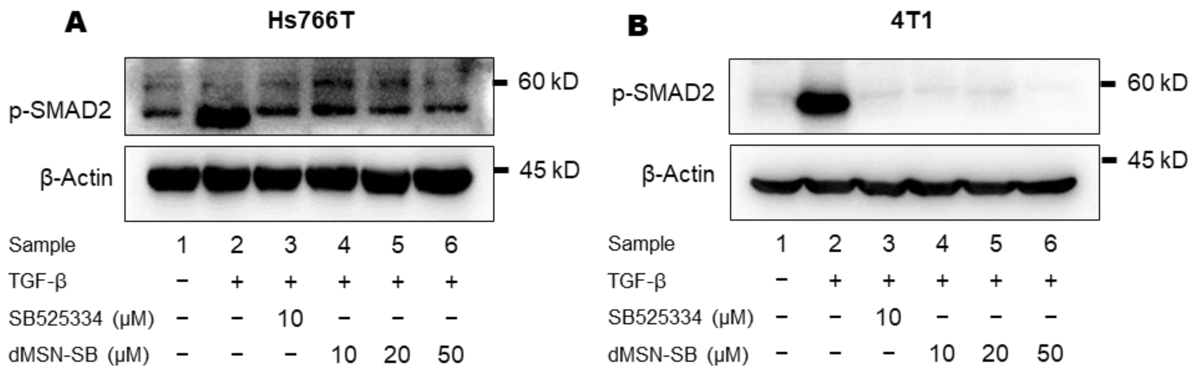

Figure S2. Expression of p-SMAD2 in Hs766T human pancreatic cancer cell line (A) and 4T1 murine breast cancer cell line (B).

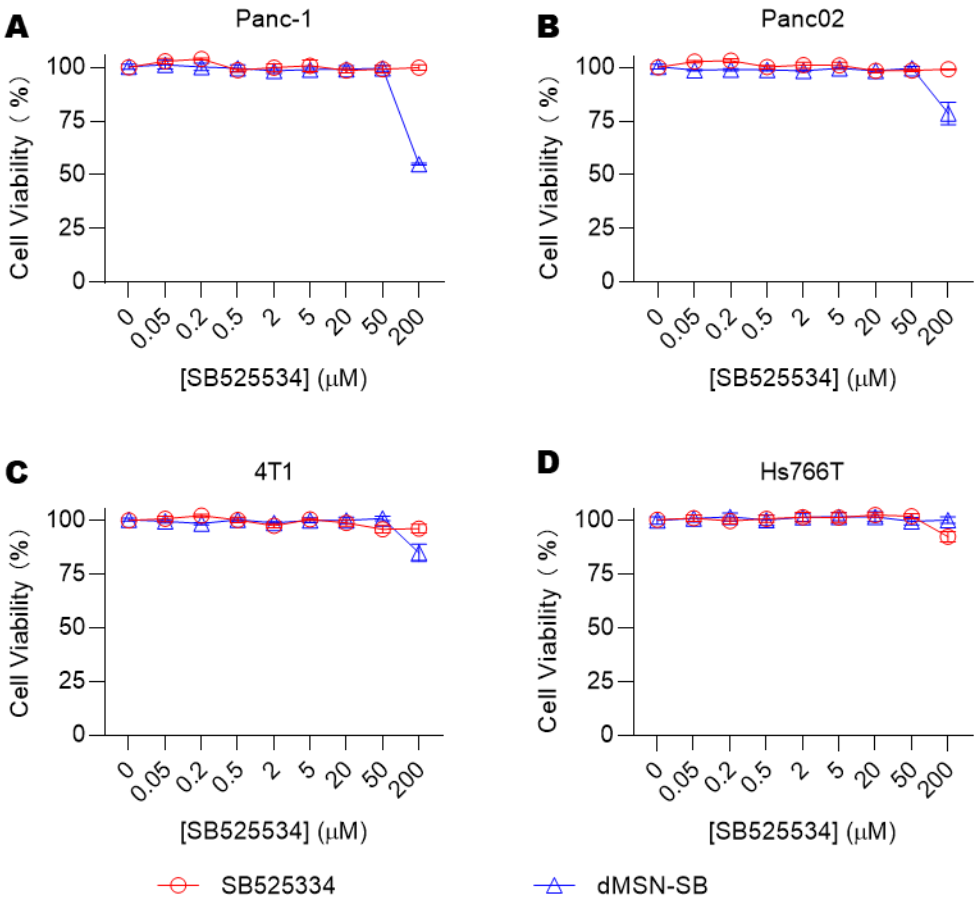

Figure S3. In vitro toxicity of dMSN-SB and SB525334. A) Panc-1 human pancreatic cancer cells; B) Panc02 murine pancreatic cancer cells; C) 4T1 murine breast cancer cells, and D) Hs766T human pancreatic cancer cells. Cells were incubated at 37 °C for 24 hours with dMSN-SB or SB525334 at concentrations equivalent to 0 to 200 μM of SB525334. Cell viability was measured using CCK-8 assay and normalized to that of untreated control. Data are shown as mean ± SEM, n = 6. Small error bars covered by the symbol are not shown.

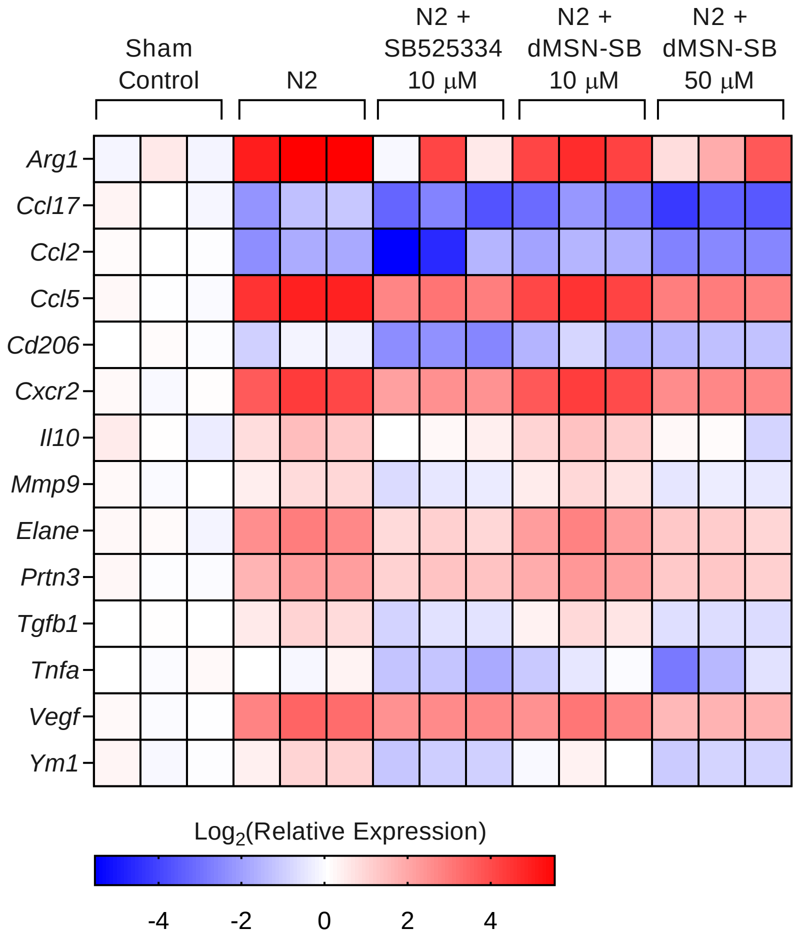

Figure S4. Relative expression of N2-associated genes in murine bone marrow-isolated neutrophils after treatment with N2 cocktail, SB525534 (10 µM), dMSN-SB (10 µM) and/or dMSN-SB (50 µM). Cells were cultured for 24 hours before analyses. Data are shown as heat map with a colored scale. Each square represents an individual independent data point. Three replicates were included in each group.

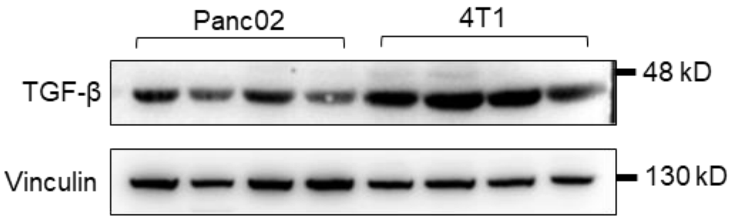

Figure S5. TGF-β expression in Panc02 and 4T1 xenograft tumors. Vinculin was used as loading control. Four independent tumors were analyzed for each tumor type.

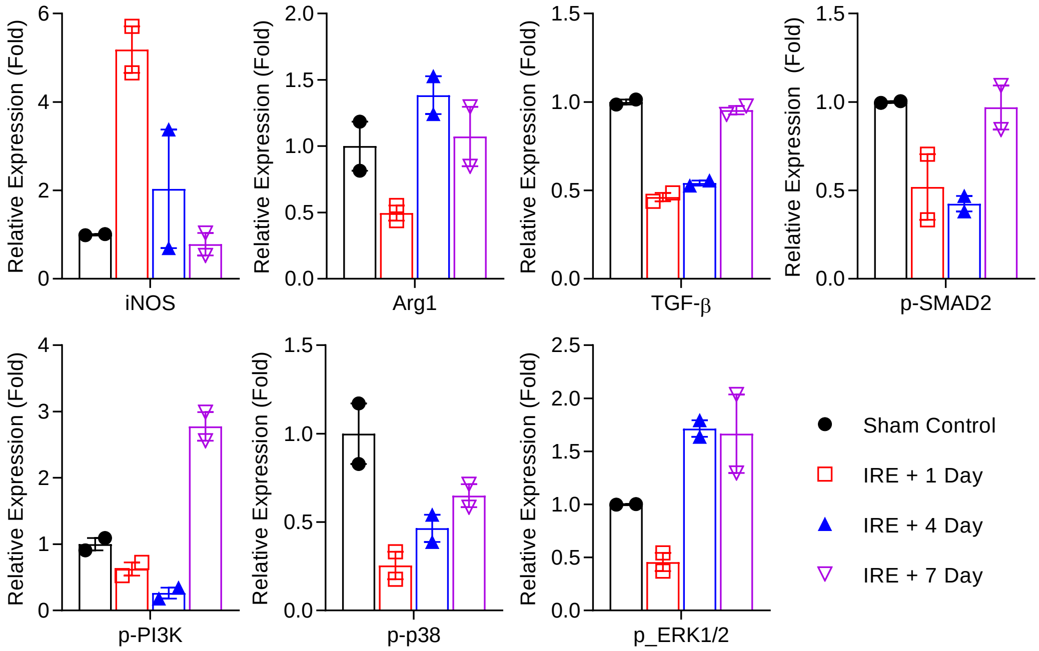

Figure S6. Quantification of immunoblots in Figure 2E. Signal intensity from each band was integrated and normalized to those of β-actin in sham control. Data are presented as mean ± SEM of *n* = 2 in bar graphs overlaid with individual data points

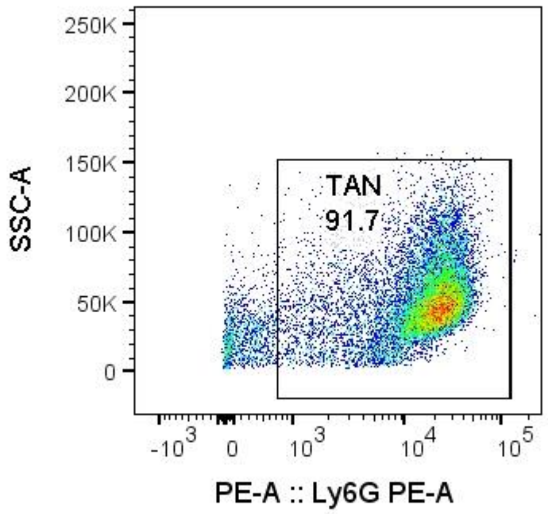

Figure S7. Flowcytometry analysis of TAN puritiy after magentic activated cell sorting.

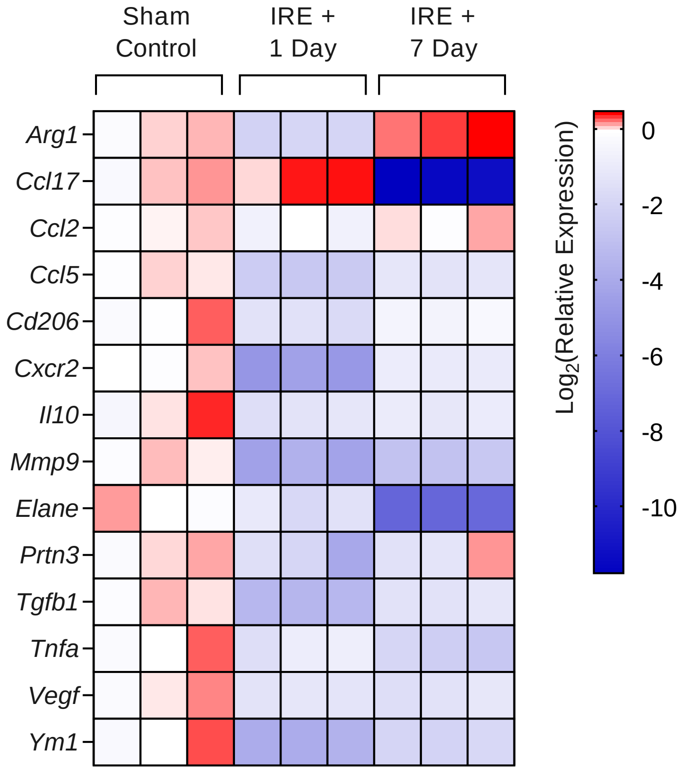

Figure S8. RT-PCR analyses of N2-associated genes in TANs isolated from Panc02 tumors post-IRE. Data are shown as heat map with a colored scale. Each square represents an individual independent data point. Three replicates were included in each group.

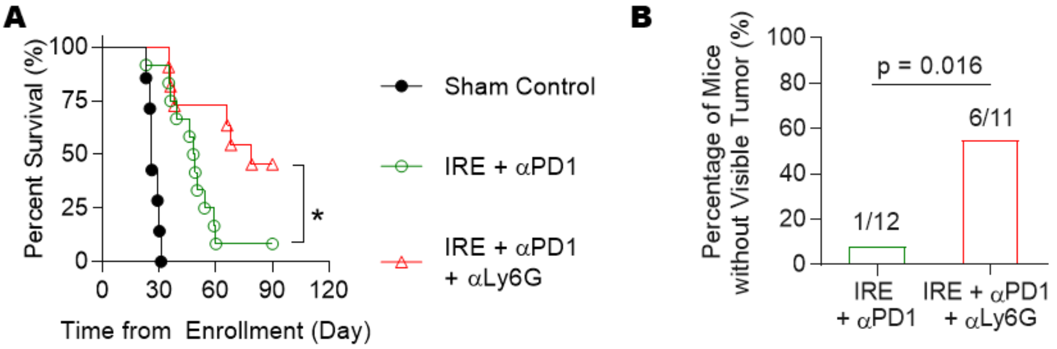

Figure S9. Effect of neutrophil depletion on the efficacy of IRE + αPD1 treatment in subcutaneous Panc02 model. A) Kaplan-Meier survival curves of sham control (black solid circle, n = 7), IRE + αPD1 (*n* = 12), and IRE + αPD1 + αLy6G (*n*= 11). Survival difference in was evaluated using Log-rank test, *$p < 0.05$. B) Percentage comparison of mice without visible tumors on day 90 after enrollment. IRE + αPD1 (1 out of 12) or IRE + αPD1 + αLy6G (6 out of 11). Significance of difference was examined using $\chi 2$ test.

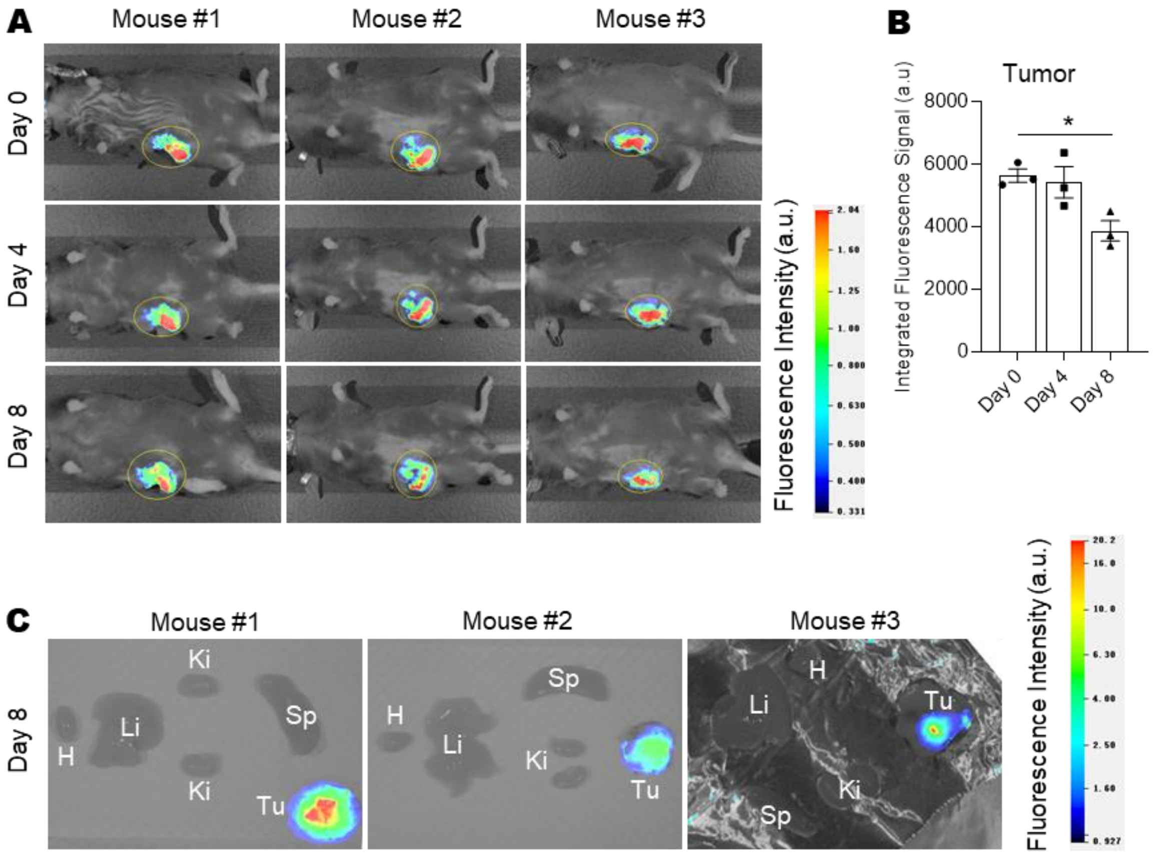

Figure S10. Fluorescence tracking of dMSN-SB after intratumoral injection. A) Whole body fluorescence imaging of Panc02 tumor at different time points after IRE. B) Quantification of integrated fluorescence signals of tumor regions. C) Fluorescence imaging of different organs on day 8 after IRE. Experimental procedure: Near-infrared fluorescence dye DiR was loaded during preparation of dMSN-SB, and the resultant formulation was intratumoral injected after IRE using the same dose for therapy studies. The mice were imaged on Pearl Trilogy Imaging System (Li-Cor Biosciences, NE, USA). Data are presented as mean ± SEM in bar graphs overlaid with individual data points. Significance was determined using one-way ANOVA followed by Tukey post hoc analysis. *$p < 0.05$.

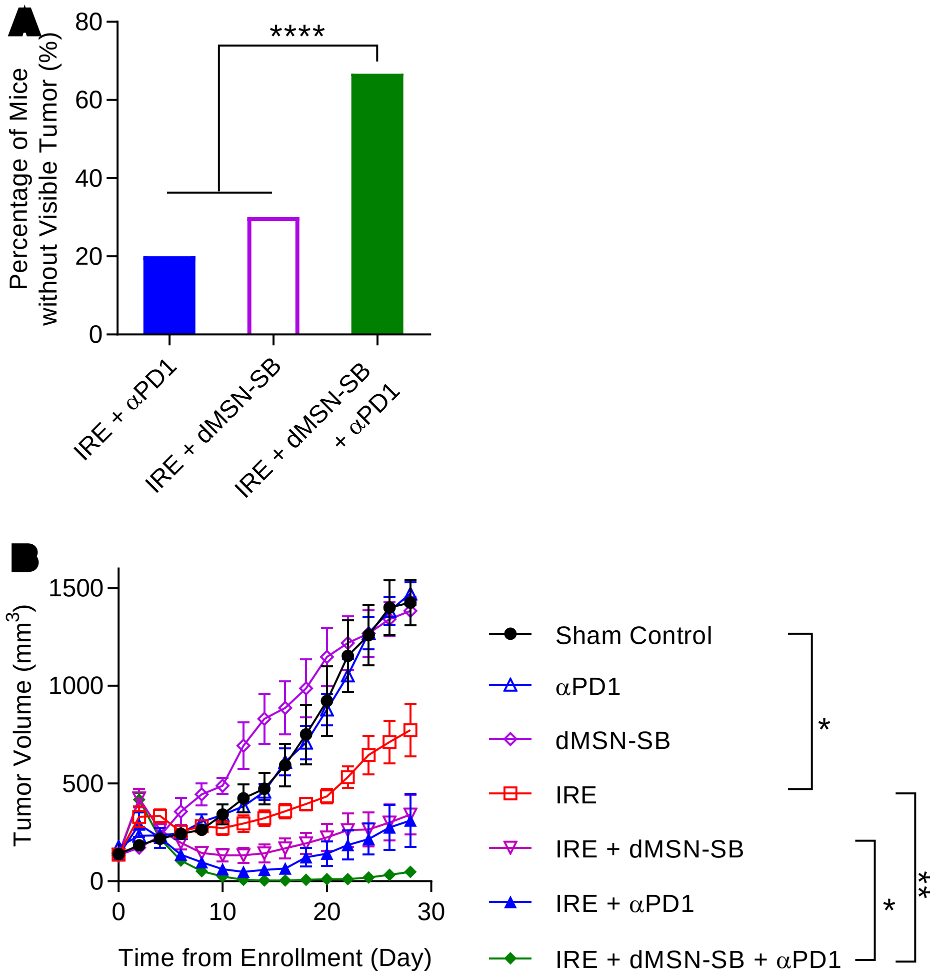

Figure S11. Anti-tumor efficacy in subcutaneous Panc02 model. A) Percentage comparison of mice without visible on day 90 after enrollment in groups of IRE + αPD1 (20%), IRE + dMSN-SB (30%), and IRE + dMSN-SB + αPD1 (67%). Significance of difference was examined using $\chi 2$ test. B) Tumor growth curves up to 28 days after enrollment in groups of sham control (n = 5), IRE (n = 5), dMSN-SB (n = 7), αPD1 (n = 5), IRE + αPD1 (n = 5), IRE + dMSN-SB (n = 10), IRE + dMSN-SB + αPD1 (n = 15). Data are shown as mean ± SEM in symbols. Significance of differences was determined using one-way ANOVA followed by Tukey post-hoc analysis. *$p < 0.05$, **$p < 0.01$.

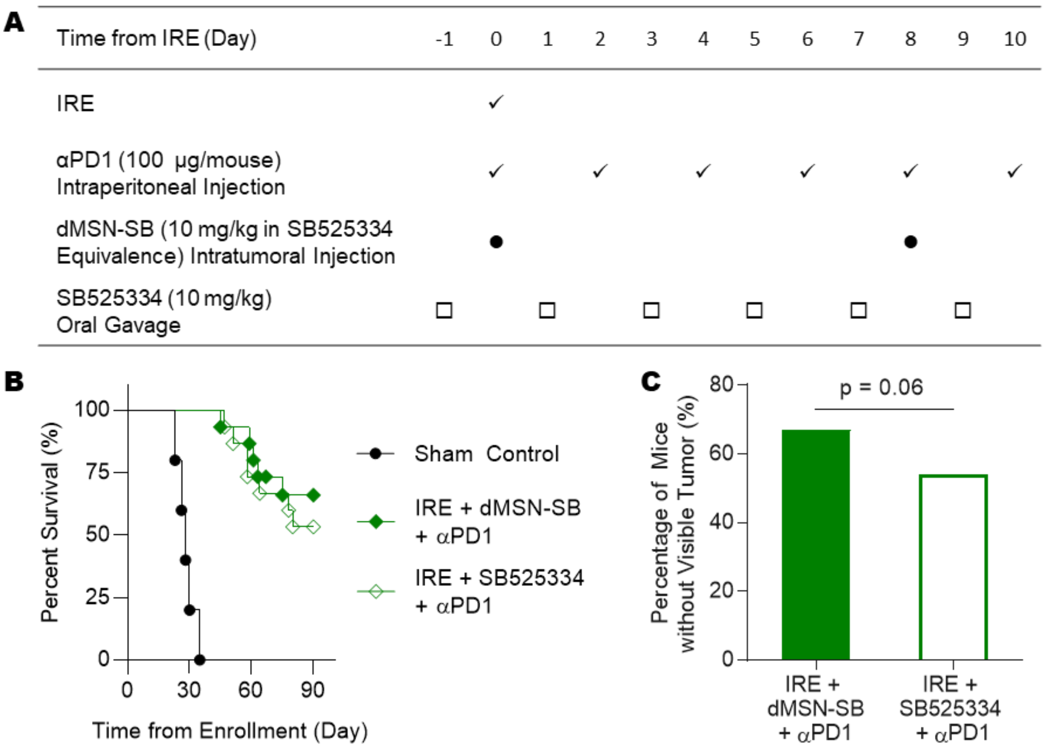

Figure S12. Comparision between dMSN-SB and unloaded SB525334 for enhancment of IRE + αPD1 therapy. A) Treatment dosage, routes, and schedules for subcutaneous Panc02 tumor model. B) Kaplan-Meier survival cuves of sham control (n = 5), IRE + dMSN-SB + αPD1 (n = 15), and IRE + SB525334 (n = 15). C) Percentage comparison of mice with no visible tumor on day 90, p = 0.06, χ2-test.

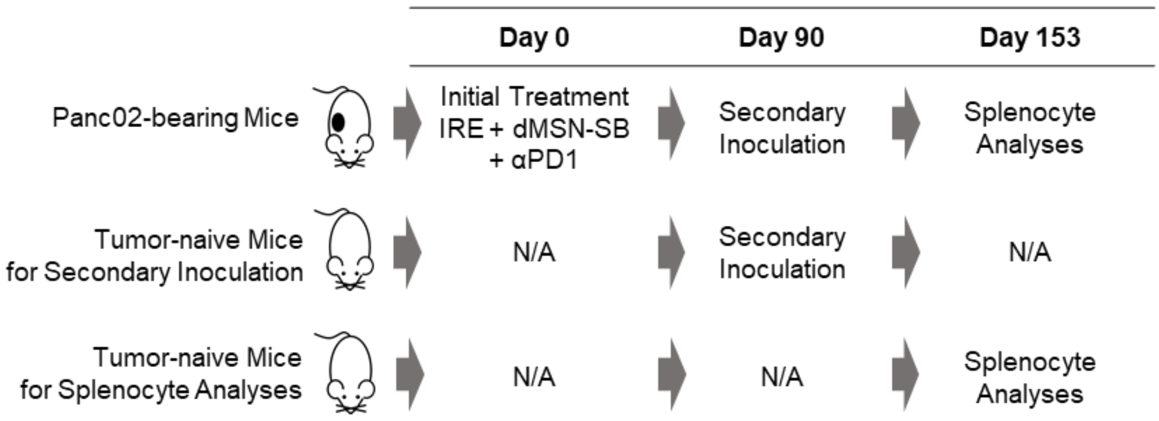

Figure S13. Timeline for re-challenge study and splenocyte analyses.

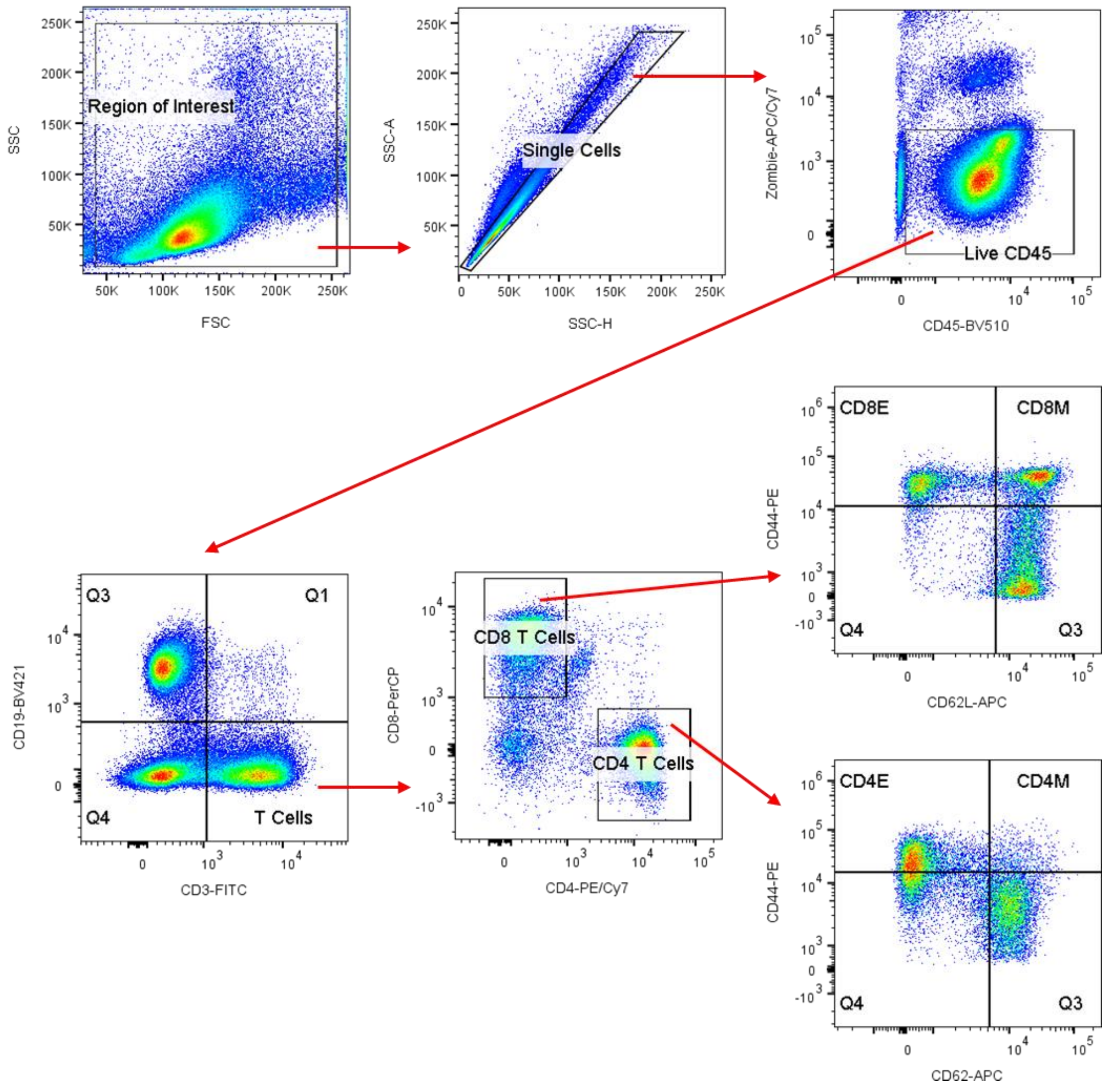

Figure S14. Gating strategy for memory splenocytes.

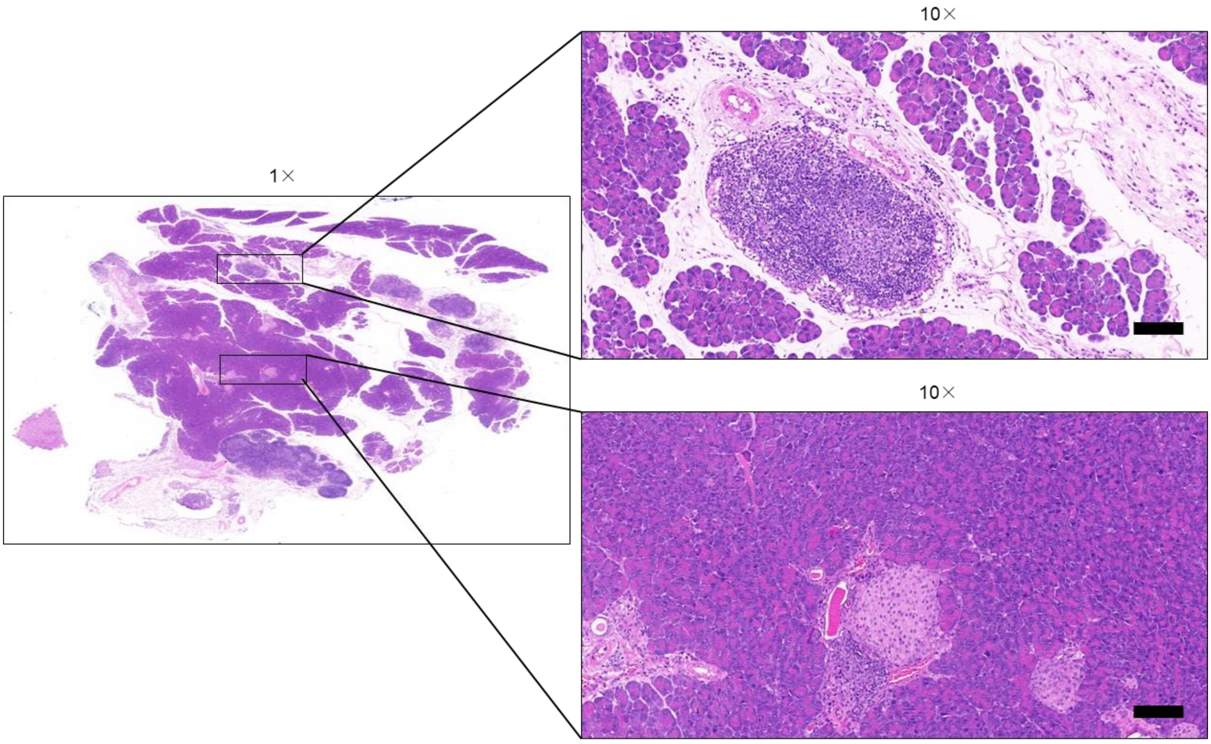

Figure S15. Representative H&E staining of tumor-free pancreas in cured orthotopic Panc02 model. Scale bar = 100 μm.

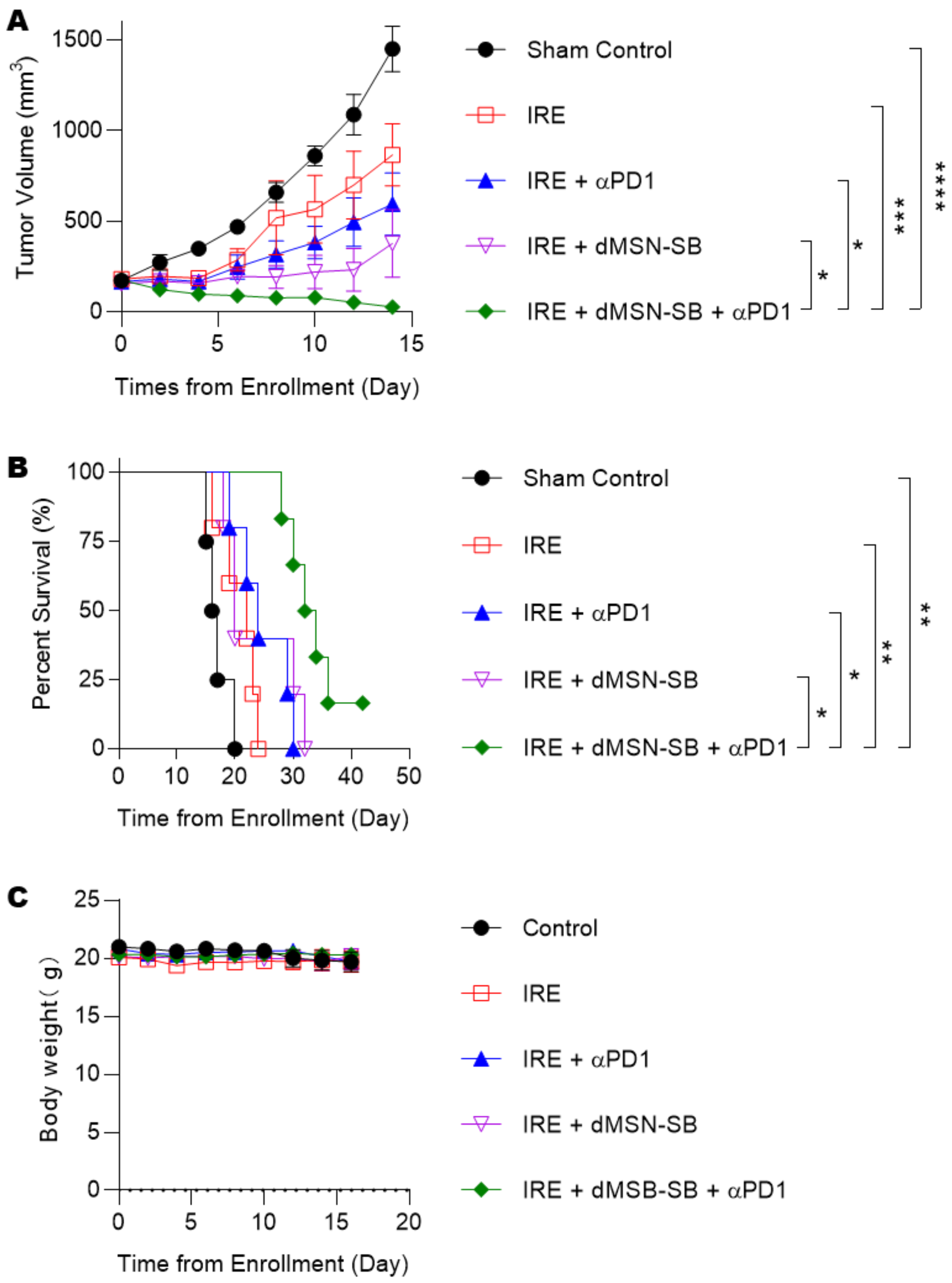

Figure S16. Anti-tumor efficacy in murine 4T1 orthotopic breast tumor model. A) Tumor growth curves to 15 days after start of treatments. B) Kaplan-Meier survival cures. C) Changes of body weight during treatment.

A

Time from IRE (Day)	0	1	2	3	4	5	6	7
IRE	✓							
αPD1 (100 μg/mouse) Intraperitoneal Injection	✓		✓		✓		✓	Collect Tumor
dMSN-SB (10 mg/kg in SB525334 Equivalence) Intratumoral Injection	●							

B

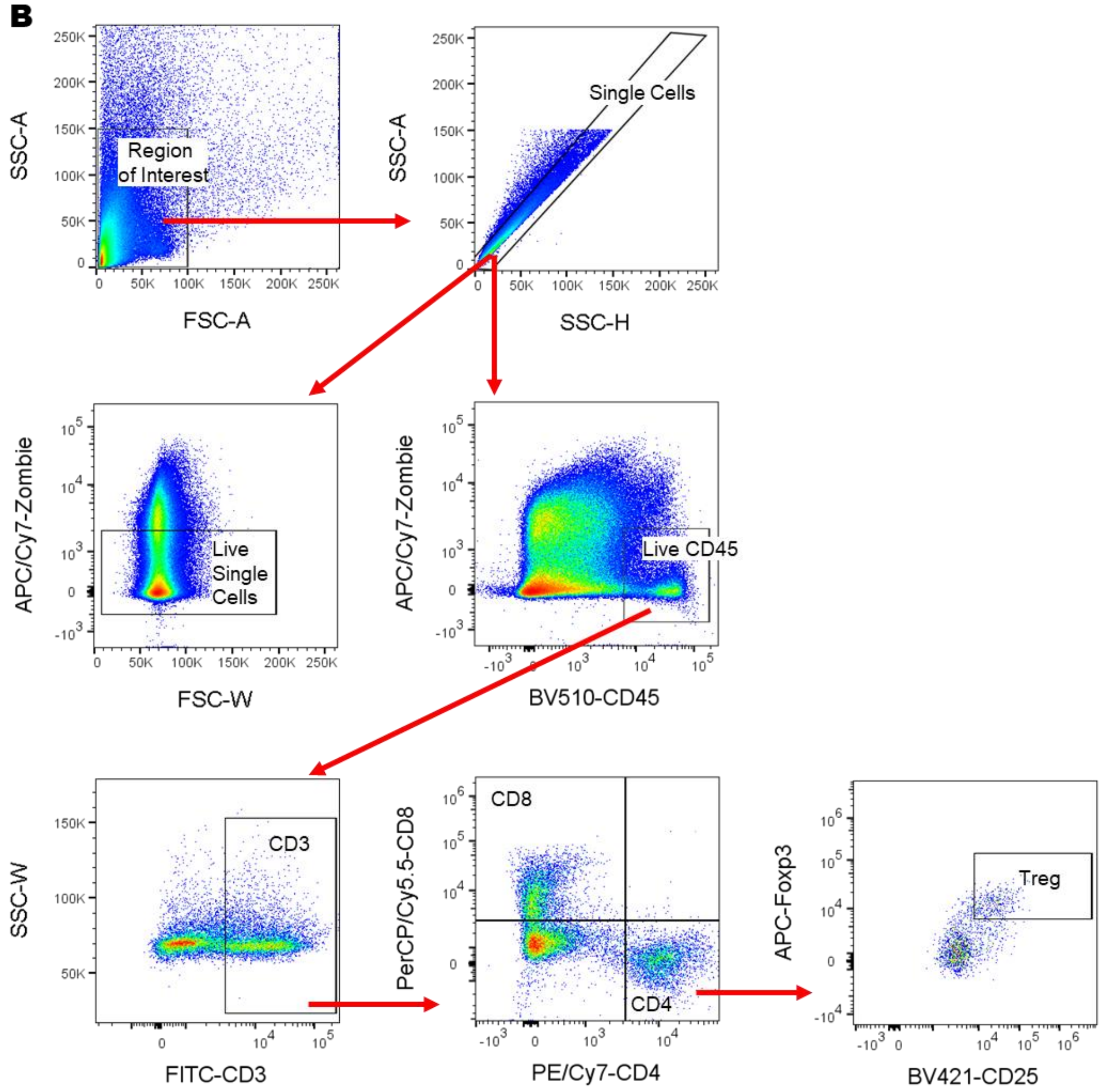

Figure S17. Gating strategy for intratumoral T cell flowcytometry. A) Schedule for treatment and tumor collection. B) Gating strategy for T cells.

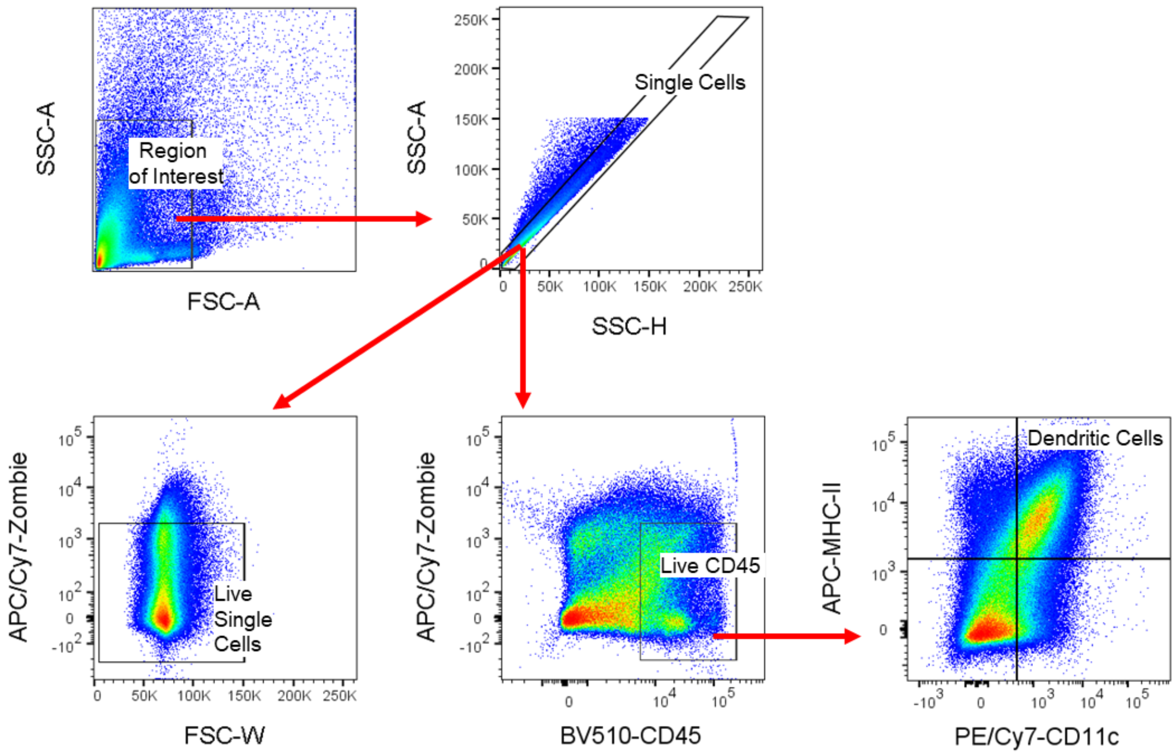

Figure S18. Gating Strategy for intratumoral dendritic cells

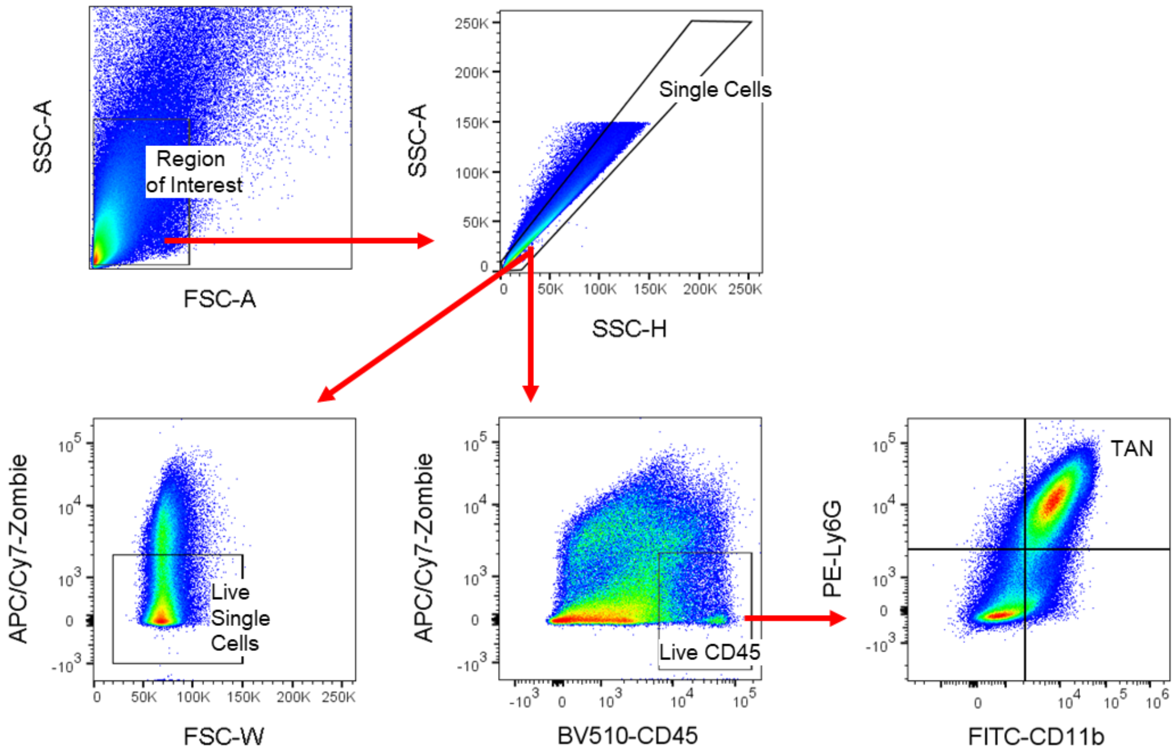

Figure S19. Gating Strategy for TANs

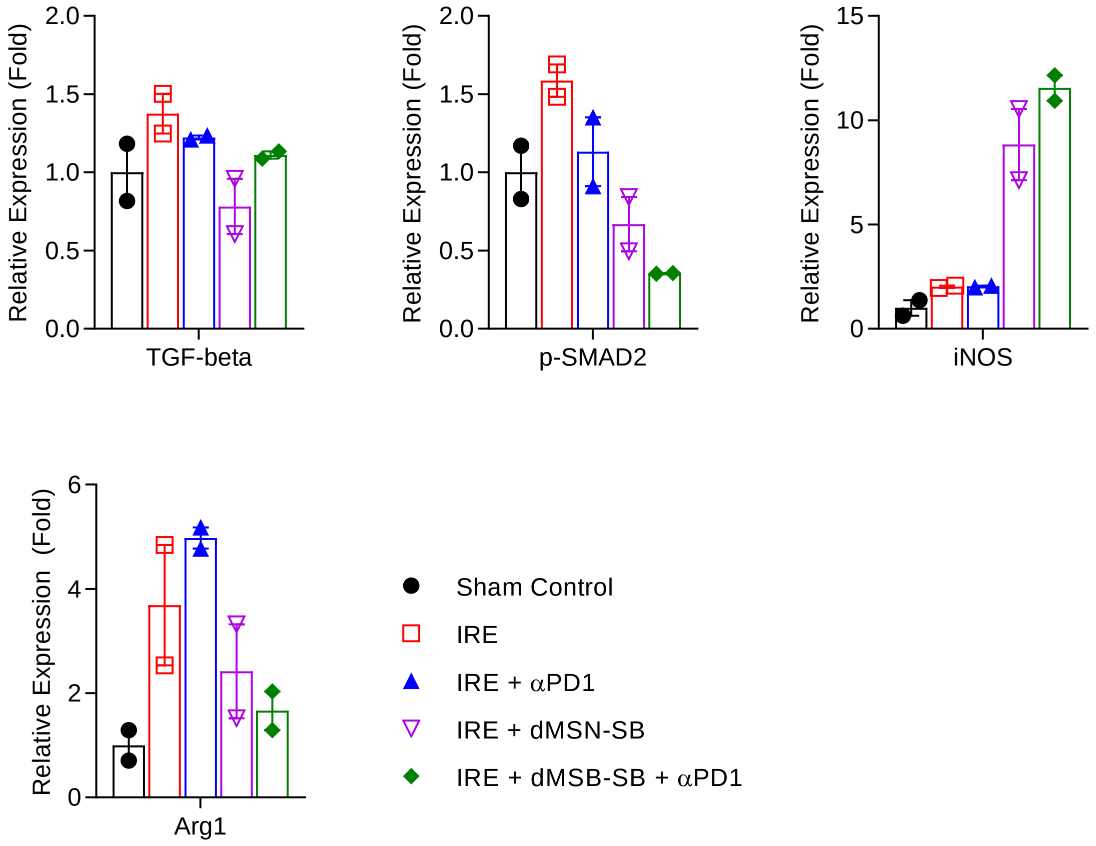

Figure S20. Quantification of Immunoblots in Figure 6A. Signal intensity from each band was integrated and normalized to those of β-actin in sham control.

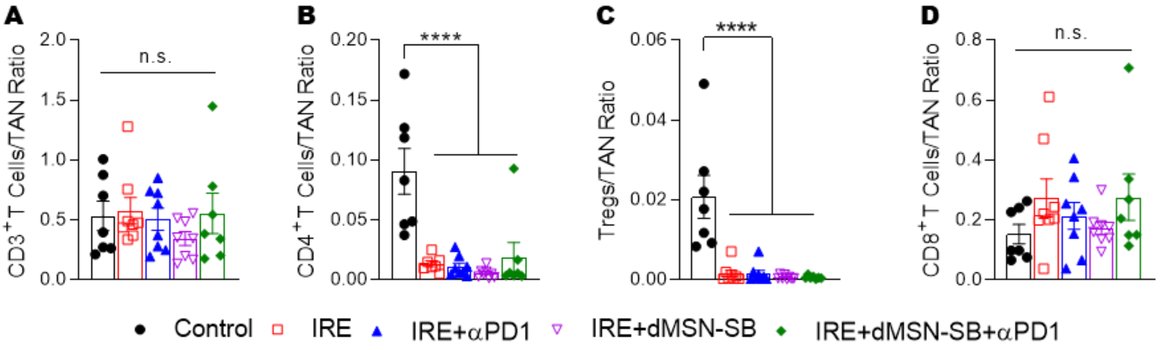

Figure S21. Relative abundance of T cell subpopulations to that of TAN. Significance of differences was determined using one-way ANOVA followed by Tukey post-hoc analysis. ****$p < 0.0001$,

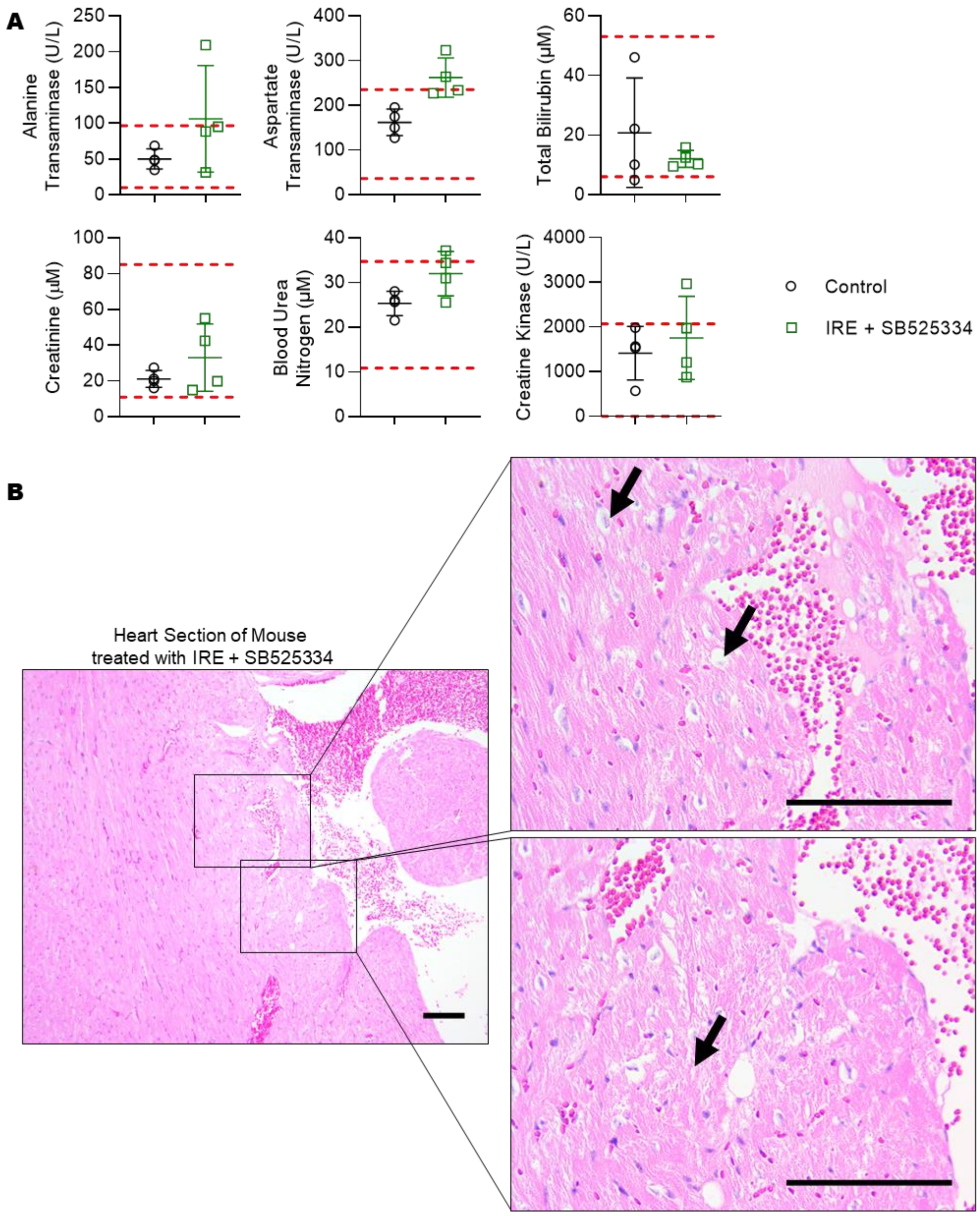

Figure S22. Toxicology studies on Panc02-bearing mice treated with IRE + SB525334. A) Blood chemistry analyses of liver, kidney, and heart toxicity in mice of sham control (n = 4) or IRE + SB252334 group (n = 4). The upper and lower limits of normal ranges are marked with red dashed lines. B) H&E staining of heart section in one mouse that showed abnormal blood chemistry results after IRE + SB525334 treatment. Regions of hydropic degeneration, fatty degeneration, and myolysis were observed (black arrows). Scale bar = 100 μm. Mice were treated with IRE on day 0, and administered with SB525334 at 10 mg kg^{-1} per dose via oral gavage on days -1, 1, 3, 5, 7, and 9. Blood and tissue samples were collected on day 10 and then analyzed. Four mice were included in each group. Untreated mice were used as control.

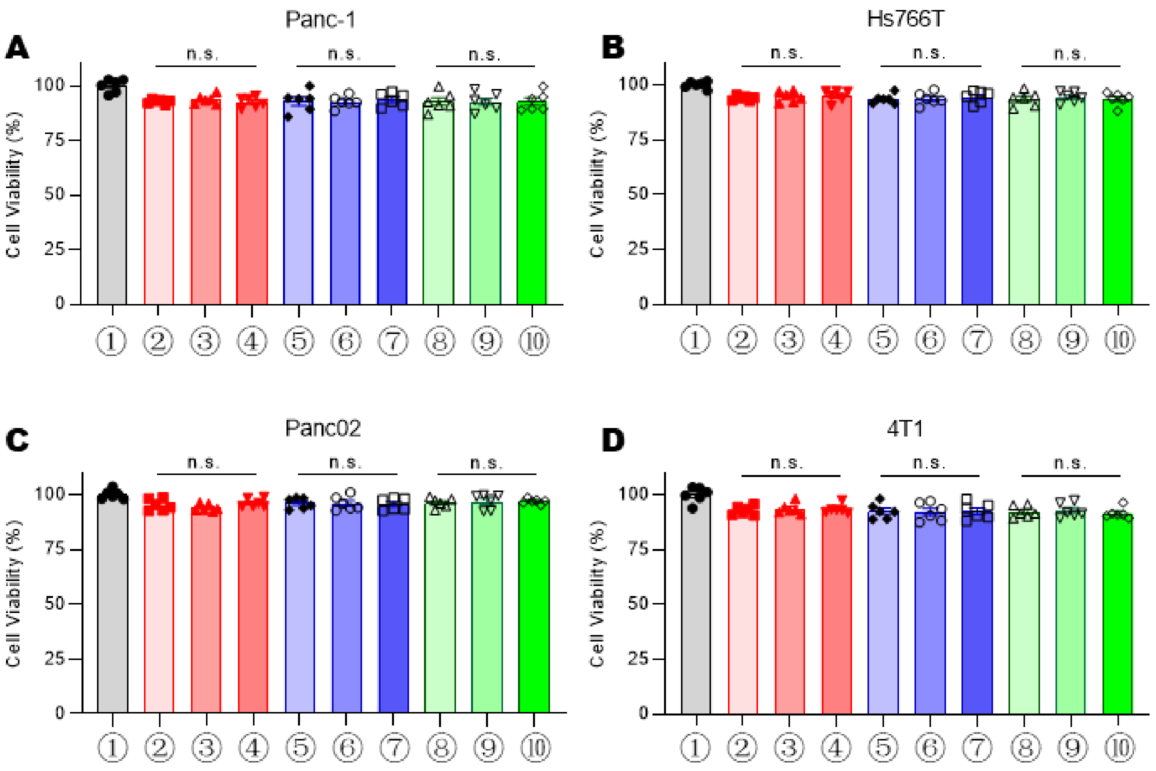

E

Sample ID	①	②	③	④	⑤	⑥	⑦	⑧	⑨	⑩
SB525334 (μM)	0	0	10	50	0	10	50	0	10	50
Pulse Intensity (V/cm)	0	200			400			600		

Figure S23. Cell viability after treatment with reversible electroporation and/or SB525334. A&B) human pancreatic cancer cell lines Panc-1 and Hs766T; C) murine pancreatic cancer cell line Panc02; D) murine breast cancer cell line 4T1. E) Layout of samples and corresponding experimental conditions. Electroporation parameters: gap between electrodes: 4 mm; pulse duration: 100 μs; interval between pulses: 1 s; number of pulses: 20; pulse voltage: 80, 160, and 120 V. Panc-1 cells were suspended at 2.0 × 10^6/mL in complete medium, and subjected to electroporation in a cuvette. The cells were then seeded at 1.0 × 10^4/well in a 96-well plate with SB525334 in complete medium, and cultured for 24 h. Cell viability was measured via CCK-8 assay. Six replicates were included in each group. Data was presented as mean ± SEM. Statistical significance was determined using one-way ANOVA.

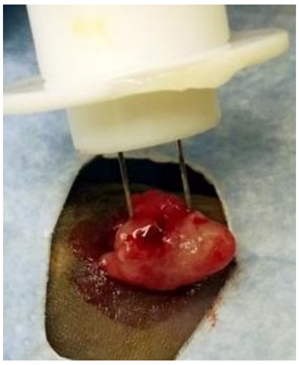

Figure S24. Photograph of IRE-treatment on orthotopic pancreatic tumor model.

Table S1. Gene ontology analysis based on 11 upregulated cytokines in Figure 7D

Analysis Type: PANTHER Overrepresentation Test (Released 20210224)<br>Annotation Version and Release Date:<br>GO Ontology database DOI: 10.5281/zenodo.5228828 Released 2021-08-18<br>Analyzed List: upload_1 (Mus musculus)<br>Reference List: Mus musculus (all genes in database)<br>Test Type: FISHER<br>Correction: FDR			
**GO Biological Process**	**Fold Enrichment**	**Raw P-Value**	**FDR**
leukocyte migration (GO:0050900)	81.77	6.62E-17	1.04E-12
leukocyte chemotaxis (GO:0030595)	> 100	4.76E-16	3.75E-12
cytokine-mediated signaling pathway (GO:0019221)	59.18	1.14E-15	6.00E-12
cell chemotaxis (GO:0060326)	76.51	1.27E-14	4.99E-11
inflammatory response (GO:0006954)	38.28	5.40E-14	1.70E-10
neutrophil chemotaxis (GO:0030593)	> 100	9.39E-13	2.11E-09
positive regulation of immune system process (GO:0002684)	18.54	8.85E-13	2.32E-09
granulocyte chemotaxis (GO:0071621)	> 100	1.57E-12	2.74E-09
cellular response to cytokine stimulus (GO:0071345)	26.34	1.49E-12	2.93E-09
neutrophil migration (GO:1990266)	> 100	2.35E-12	3.70E-09
positive regulation of response to external stimulus (GO:0032103)	37.54	3.37E-12	4.83E-09
granulocyte migration (GO:0097530)	> 100	4.64E-12	6.09E-09
response to cytokine (GO:0034097)	22.95	5.07E-12	6.14E-09
chemotaxis (GO:0006935)	31.98	1.19E-11	1.34E-08
taxis (GO:0042330)	31.67	1.29E-11	1.35E-08
response to external biotic stimulus (GO:0043207)	13.52	2.01E-11	1.59E-08
response to other organism (GO:0051707)	13.54	1.99E-11	1.65E-08
cell migration (GO:0016477)	20.01	1.71E-11	1.68E-08
myeloid leukocyte migration (GO:0097529)	94.44	1.96E-11	1.72E-08
defense response (GO:0006952)	13.62	1.88E-11	1.74E-08
leukocyte migration (GO:0050900)	81.77	6.62E-17	1.04E-12

[1]Top 30 enriched biological processes are listed

Table S2. Primer sequences

Murine			
Target		Sequence (5' to 3')	Refences
Ccl3	Forward	TTCTCTGTACCATGACACTCTGC	PrimerBank ID 6755432a1
	Reverse	CGTGGAATCTTCCGGCTGTAG	
Cxcl9	Forward	AACCTCCCACGTAGCTTTCG	NM_008599.4
	Reverse	GCCAATGCCTGGTGTGTAAC	
Fas	Forward	TATCAAGGAGGCCCATTTTGC	XM_030250751.2
	Reverse	TGTTTCCACTTCTAAACCATGCT	
Icam1	Forward	CTGCAGACAGTGACCATC	NM_008625.2
	Reverse	GTCCAGTTTCCCGGACAA	
Ifng	Forward	ATGAACGCTACACACTGCATC	NM_008337.4
	Reverse	CCATCCTTTTGCCAGTTCCTC	
Nos2	Forward	GTTCTCAGCCCAACAATACAAGA	PrimerBank ID 6754872a1
	Reverse	GTGGACGGGTCGATGTCAC	
Arg1	Forward	CTCCAAGCCAAAGTCCTTAGAG	PrimerBank ID 7103255a1
	Reverse	AGGAGCTGTCATTAGGGACATC	
Ccl17	Forward	AATGTAGGCCGAGAGTGCTG	NM_011332.3
	Reverse	TGCCCTGGACAGTCAGAAAC	
Ccl2	Forward	CAGATGCAGTTAACGCCCCA	NM 011333.3
	Reverse	TGAGCTTGGTGACAAAAACTACAG	
Ccl5	Forward	TGCTGCTTTGCCTACCTCTC	NM 013653.3
	Reverse	TCCTTCGAGTGACAAACACGA	
Cd206	Forward	GATCCTCAACCCAAGGGCTC	NM_008625.2
	Reverse	ACCAATGCAACCCAGTGCTA	
Cxcl10	Forward	CCAAGTGCTGCCGTCATTTTC	NM_021274.2
	Reverse	GGCTCGCAGGGATGATTTCAA	
Cxcr2	Forward	GCTCACAAACAGCGTCGTAG	NM_009909.3
	Reverse	CCATGCTGATGCAGGCTAGT	
Il10	Forward	AGCCTTATCGGAAATGATCCAGT	NM_010548.2
	Reverse	GGCCTTGTAGACACCTTGGT	
Mmp9	Forward	GCAGAGGCATACTTGTACCG	NM_013599.5
	Reverse	CGTCGTCGAAATGGGCATCT	
Neutrophil Elastase	Forward	CATCTGCTTCGGGGACTCTG	NM_015779.2
	Reverse	CCCTCTCGGTCTTTGGGATG	
Prtn3	Forward	CACCTTCCTATGCCGGGAAC	NM_011178.2
	Reverse	CCGCAGCACGTTTTGAATCC	
Tgfb1	Forward	CTCCCGTGGCTTCTAGTGC	PrimerBank ID 6755775a1
	Reverse	GCCTTAGTTTGGACAGGATCTG	
Tnfa	Forward	GACGTGGAACTGGCAGAAGAG	NM_001278601.1
	Reverse	TTGGTGGTTTGTGAGTGTGAG	
Vegf	Forward	GCACATAGAGAGAATGAGCTTCC	PrimerBank ID

	Reverse	CTCCGCTCTGAACAAGGCT	6678563a1
Ym1	Forward	TTTGGACCTGCCCCGTTC	NM_009892.3
	Reverse	CCTTGGAATGTCTTTCTCCACA	

[1]Primers are selected from PrimerBank or designed with Primers 3.0.

Table S3. List of antibodies used for flowcytometry

Antibody	Manufacturer	Clone	Catalogue Number
CD11b-FITC	Biolegend	M1/70	101205
CD11c-PE/Cyanine7	Biolegend	N418	117317
CD19-Brilliant Violet 421	Biolegend	6D5	115537
CD206-Alexa Fluor647	Biolegend	C068C2	141711
CD25-Brilliant Violet 421	Biolegend	PC61	102043
CD3ε-FITC	Biolegend	145-2C11	100305
CD44-PE	Biolegend	IM7	103007
CD45-Brilliant Violet 510	Biolegend	30-F11	103138
CD4-PE/Cyanine7	Biolegend	GK1.5	100421
CD54-PE/Cyanine7	Biolegend	YN1/1.7.4	116121
CD62L-APC	Biolegend	MEL-14	104411
CD80-PerCP/Cyanine5.5	Biolegend	16-10A1	104721
CD86-PE	Biolegend	A17199A	159203
CD8a-PerCP	Biolegend	53-6.7	100731
CD95-PerCP/Cyanine5.5	Biolegend	SA367HB	152609
F4/80-PE	Biolegend	BM8	123109
FOXP3-Alexa Fluor 647	Biolegend	MF-14	126407
I-A/I-E-APC	Biolegend	M5/114.15.2	107613
Ly6C- Brilliant Violet 421	Biolegend	HK1.4	128031
Ly6G/Ly6C-PerCP	Biolegend	RB6-8C5	108425
NK1.1-PE	Biolegend	PK136	108707

Table S4. List of antibodies used for immunoblotting

Antibody	Manufacturer	Catalogue Number	LOT
β-actin	Abclonal	AC006	9100006002

iNOS	Absin	abs130136	MF12
p-Erk 1/2 (Thr202/Tyr204)	Absin	abs130614	SD09
p-PI3K (Tyr607)	Absin	abs130868	JB11
p-P38 (Thr180/Tyr182)	Absin	abs131122	AI08
TGF-β	Cell signaling	3711S	7
Arginase-1	Cell signaling	936681	1
p-Smad2	Cell signaling	3108T	8

Table S5. Target list of mouse cytokine array

	1, 2	3, 4	5, 6	7, 8	9, 10	11,12	13, 14	15, 16	17, 18	19, 20	21, 22	23, 24
A	Control											Control
B	CXCL13	C5a	G-CSF	GM-CSF	CCL1	CCL11	ICAM1	IFN-γ	IL1α	IL-1β	IL1-ra	IL-2
C	IL-3	IL-4	IL-5	IL-6	IL-7	IL-10	IL-13	IL-12p70	IL-16	IL-17	IL-23	IL-27
D	CXCL10	CXCL11	CXCL1	M-CSF	CCL2	CCL12	CXCL9	CCL3	CCL4	MIP-2	CCL5	CXCL12
E	CCL17	TIMP-1	TNF-α	TREM-1								
F	Control											

Table S6. List of antibodies used for immunohistochemical staining

**Antibody**	**Manufacturer**	**Catalogue Number**	**LOT**
Ki67	Servicebio	GB111499	LS203021
MPO	Servicebio	GB11224	AC2104004E
Ly-6G	Servicebio	GB11229	AC2103022C
